# Recent approaches and advancement in biochar-based environmental sustainability: Is biochar fulfilling the sustainable development goals?

**DOI:** 10.1016/j.isci.2024.110812

**Published:** 2024-09-07

**Authors:** Ashmita Patro, Saurabh Dwivedi, Anjali Thakur, Prafulla Kumar Sahoo, Jayanta Kumar Biswas

**Affiliations:** 1 Academy of Scientific and Innovative Research (AcSIR), Ghaziabad 201002, India; 2 CSIR-Institute of Minerals and Materials Technology, Bhubaneswar 751013, Odisha, India; 3 Department of Environmental Science and Technology, Central University of Punjab, V.P.O. Ghudda, Bathinda 151401, Punjab, India; 4 Department of Ecological Studies and International Centre for Ecological Engineering, University of Kalyani, Kalyani, Nadia 741235, West Bengal, India

**Keywords:** Soil science, Environmental management, Soil chemistry, Soil biology

## Abstract

This review highlights the application of biochar (BC) for attaining different SDGs (SDG 6: clean water and sanitation, SDG 7: affordable and clean energy, SDG 13: climate action, and SDG 15: life on land). These goals coincide with the various existing environmental problems including wastewater treatment, soil amendment, greenhouse gas remediation, and bioenergy generation. So, the review encompasses the various mechanisms involved in the BC-assisted treatment and reclamation of water, pollutant immobilization and enhancing soil properties, reduction of greenhouse gas emission during the wastewater treatment process and soil amendment mechanisms, bioenergy generation through various electrode material, biodiesel production, and many more. The review also explains the various drawbacks and limitations of BC application to the available environmental issues. Conclusively, it was apprehended that BC is an appropriate material for several environmental applications. More research interventions are further required to analyze the applicability of different BC materials for attaining other available SDGs.

## Graphical abstract

**Figure:**
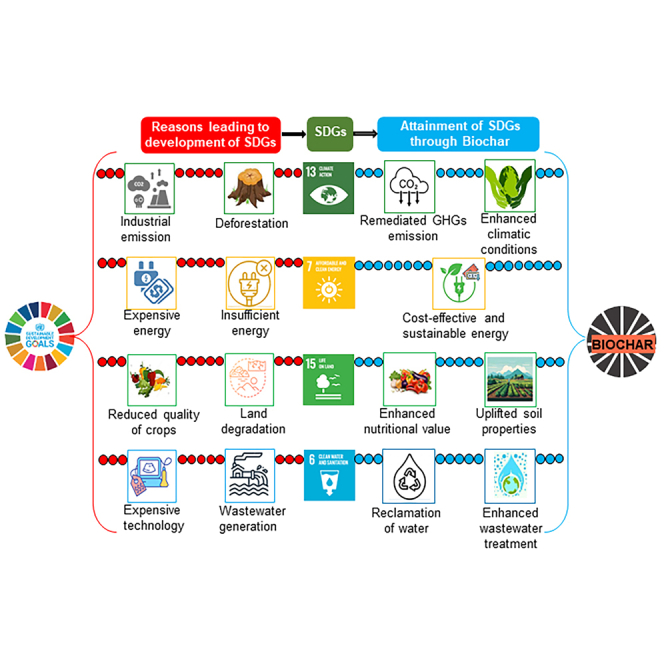

## Introduction

In the past decades, the occurrence of industrial revolution has brought vast changes in the lifestyle of human beings that adversely affected the environment. Urbanization, industrialization, and widespread chemical applications in agriculture and daily life influenced climatic variations and rapid environmental pollution (water, soil, air).^1^ This led to the occurrence of deforestation, reduced sanitation, lack of drinking water access, decreasing air quality index, huge solid waste generation, reduction in soil fertility, increased soil erosion, reduction in groundwater recharge, high cases of diseases, frequent floods, and droughts. As a result, selective competition for survival and existence began between different classes of society that unveiled the unequal distribution of food, water, and natural resources. To address these socio-economic and environmental issues of society, in 2015, the General Assembly of the United Nations proposed 17 sustainable developmental goals (SDGs) and 169 targets to sustainably improve the competing human lives globally. Some of the SDGs include Clean water and sanitation (goal 6), affordable and clean energy (goal-7), climate action (goal-13), life below water (goal-14), and life on land (goal-15). These SDGs were targeted to be achieved by the year 2030.^2^ To attain these SDGs, various materials, methods, approaches, and technological developments were proposed and implemented on a massive scale. For example, to achieve SDG 6 (clean water and sanitation) which corroborates the target of enhanced wastewater treatment, numerous advanced wastewater treatment technologies and methodologies have been investigated, such as wastewater treatment plants, sewage treatment plants, effluent treatment plants, reverse osmosis, anaerobic digesters, and constructed wetlands.^3^ Incorporating such wastewater treatment technologies led to the enhanced treatment of pollutants (organic compounds and inorganic compounds) from various waste streams and produced vast amounts of clean water for reuse. Likewise, for successfully accomplishing SDG 15 (life on land), i.e., to advance the properties of soil, various physical (soil replacement and soil thermal desorption), chemical (chemical oxidation and reduction, chemical leaching) and biological (phytoremediation and microbial degradation) technologies have been experimented.^4^ Enhancing the soil properties advanced the biological lives on land as increasing the soil quality through nutrient enrichment and nutrient uptake up-scaled the nutritional value of plants. So, the surplus growth of healthy plants will simultaneously reduce the rising issues of food security and will simultaneously decrease the threat to human lives from hunger. This will simultaneously help to achieve SDG 2 i.e., zero hunger. Similarly, SDG 13 (climate action) could be been attained through advanced air pollution control equipment (cyclone separator, fabric filters, electrostatic precipitators) which favored efficient purification of noxious gases and particulate matters released into the atmosphere through different industrial residual air discharges.^5^ Additionally, SDG 7 (clean and affordable energy) has also been achieved through various costly noble metals, semiconductors, photovoltaics, thermal power plants, fuel cells/batteries, wind turbines, hydropower plants, and so forth. that produced clean energy for various applications.^6^ However, these technologies are highly energy intensive, incur excessive costs, require greater maintenance and operational costs which evolved as major drawbacks of these systems. To overcome these disadvantages, several natural and cost-effective materials and technologies have been designed such as constructed wetlands,^7^ microbial fuel cell^8^ and their integration,^9^^,^^10^^,^^11^ and so forth*.* Nevertheless, the substrate material used in these technologies played a vital role in pollution abatement.^12^ Though various types of substrate material such as gravel, graphite, and zeolite have been investigated in these systems, still major research studies have claimed biochar (BC) as a potential candidate that effectively reduced wastewater pollutants and soil contaminants, enhanced soil properties and reduced the emission of greenhouse gases.^13^^,^^14^

Recently, BC has been considered as an essential material that is used widely for various environmental applications. It is a pyrolyzed product developed from various natural and synthetic feedstock materials in the prevalence of oxygen and temperature-controlled conditions.^15^ BC possesses various physio-chemical properties depending upon the raw biomass used and the pyrolysis conditions.^16^ These influential parameters affect the characteristics of BC, e.g., the surface area, porosity of BC, functional group, long-term stability, and adsorption property.^17^ As a result, such evolved properties of BC favor the enhancement of wastewater treatment, facilitating efficient soil amendment, carbon sequestration, reduce greenhouse gas (GHG) emission (such as methane, and nitrous oxide), heavy metal bioavailability in soil and water, and bio-energy production. Modified BC has also been used to achieve many other targets especially for wastewater treatment, nutrient immobilization, and as a slow-release fertilisers.^18^^,^^19^ BC also contributes to the global nutrient cycle such as carbon, nitrogen, and phosphorus.^20^ It helps to reduce the biomass waste burden and fix the carbon for a longer period, reducing greenhouse gas emissions.^20^ Furthermore, BC production from photosynthetic/microbial biomass shows the economic benefits in terms of its reproducibility, low-cost raw material, waste reduction, easy availability, and wider environmental applications.^16^ BC also possesses some profound properties such as specific functional groups, high porosity, high cation exchange capacity, high specific surface area, and high stability, which makes it a suitable candidate for different environmental applications.^17^ Consequently, BC has been used on a larger scale for scientific studies and field-scale application as well.^21^^,^^22^^,^^23^ Interestingly, the summation of these environmental applications of BC resonates completely with some of the goals associated with SDGs (i.e., SDG 6, SGD 7, SDG 13, and SDG 15) as mentioned above. So, these assumptions ascertain BC as a suitable candidate for fulfilling several goals of SDGs. Till now, various research studies have been available that determine BC as a potential material for treating various wastewater types, remediating pollutants from soil and aqueous environment, reducing greenhouse gas emissions, and so forth. Similarly, there exist different review articles demonstrating the role of BC in such environmental applications. However, very limited review studies are available that correlate the application of BC with the available SDGs. So, the present review is designed to affirm the potential applications of BC in dealing with various environmental aspects which simultaneously corroborate well with the targets of SDGs. Moreover, the review article has accumulated different research articles available till date to justify the effective use of BC for remediating pollutants efficiently from soil, air, and water mediums and generating bioenergy for other implications. Further sections will explicitly provide insights into the mechanisms and the functions of BC taking place during these environment upliftment processes.

## Review methodology

Considering a systematic review,^24^ we have first initiated with the identification of a knowledge area followed by a research question. The formulated research question is then followed by the study protocol consisting of data extraction, analyzing the methodology, extracting results, formulating the results in text, and finally publishing the entire research and results. Based on such procedures, a mapping of experimental research regarding the application of BC for achieving SDGs has been collected and analyzed. The collected studies include BC application in the field of wastewater treatment, soil amendment, greenhouse gas reduction, and bioenergy generation. The articles were selected in January 2024 from Science Direct, Web of Science (WOS), Scopus, and Google Scholar.

**Figure 1:**
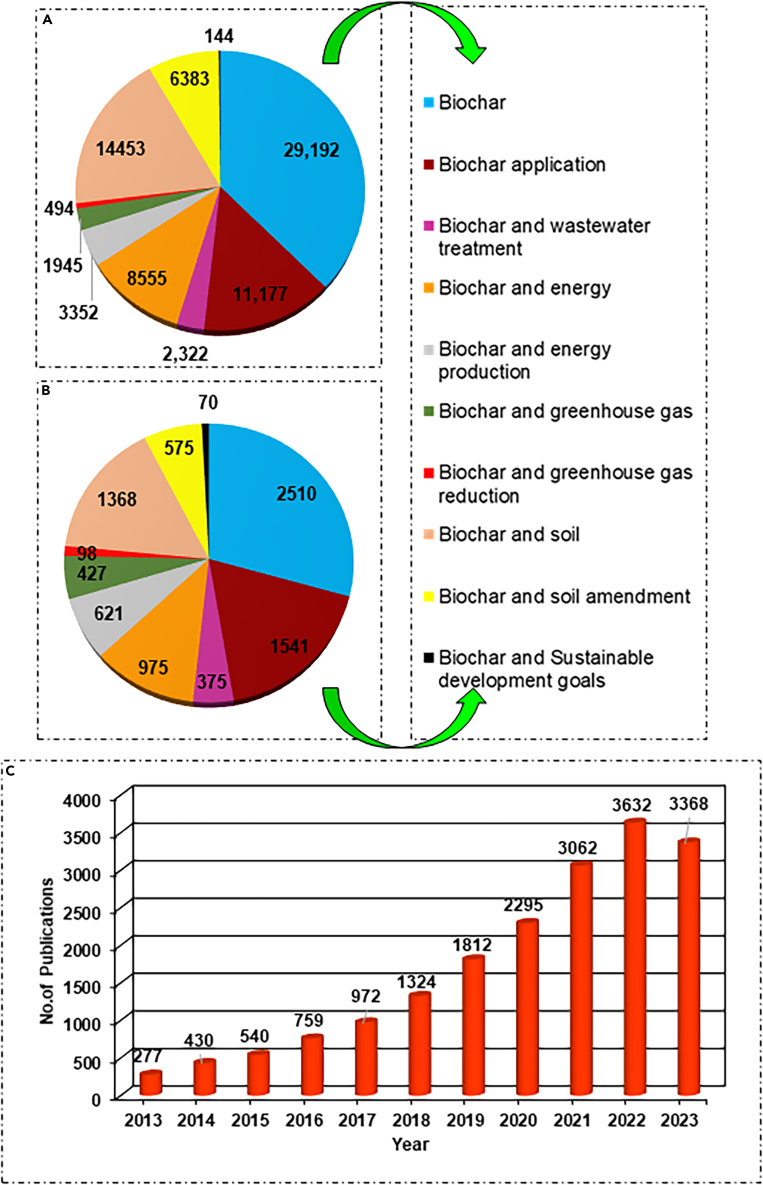
Assessment of total number of articles with respect to biochar in various environmental applications and related fields (A) Total number of review articles published; (B) Total number of articles published. The data collection was undertaken through systematic upscaling in Web of Science (https://www.webofscience.com) using search keywords, i.e., review, papers, biochar, applications, wastewater treatment, energy and production, greenhouse gas and reduction, soil and amendments, sustainable development goals. (C) Total number of research articles and review articles published on biochar during the year 2013–2023.

Following this, a research strategy has been established by providing keywords in the search platform (Web of Science) utilizing earlier research articles. The investigation of the search string was initiated by examining the terms “review,” “papers,” “biochar,” “applications,” “wastewater treatment,” “energy and production,” “greenhouse gas and reduction,” “soil and amendments,” “sustainable development goals” (as depicted in Figure 1). The search was mediated between the years 2013–2023. Around 250 articles related to BC (2000–2023) and the selected SDGs were obtained (for writing the review) and then multiple-level inclusion and exclusion criteria were applied to selectively incorporate the articles in different sections.

Further, for constructing the inclusion-exclusion approach, screening of necessary articles was carried out from the earlier collected studies. Filters were applied wherever necessary. As mentioned earlier, the review contains around 250 articles that were withdrawn since the year 2013–2023. The filtering was carried out by selectively segregating the articles based on the particular SDGs mentioned. Around 80 articles were selected for wastewater treatment, 50 articles for soil amendment, 30 articles for greenhouse gas remediation, and 20 articles for bioenergy generation. During the initial phase, screening filters were applied to the titles, keywords, and abstracts. Those 30 articles were excluded having the required title and abstract but were not suitable for the research theme. Those studies that adhere to the desired central objective of the systematic review proceeded to the next stage of selection. The last stage, where a total number of 220 articles was included were thoroughly read and further systematized for review writing. During this stage, various modulating adjustments were carried out to resolve the errors and inconsistencies obtained in the database. A full reading of each of the articles was then performed and a careful investigation of the collected articles was undertaken to design the summary. Then, the segregated articles based on the confined SDGs were presented in individual sections to clarify the actual applicability of BC in each of the determined categories. So, further sections were strategically provided based on the collected articles to accomplish the systematic review writing process relating to the thematic idea.

## Overview of biochar

Currently, the utilization of BC has been substantially extended to several environmental applications such as the qualitative treatment of wastewater, managing the generated solid waste, energy production, mitigating the prevailing climate change conditions and many more.^25^ The suitability of BC for various applications exists because of its property to act as biosorbent,^26^ photocatalyst,^27^ and so forth, which had helped in the remediation of various solid, liquid and gaseous environmental contaminants. Taking into account the applicability of BC for resolving the issues for treating all three phases of contaminants, BC has been widely used for removing various organic (dyes, pesticides, phenols) and inorganic (anions and heavy metals)^28^ pollutants from aqueous medium involving various types of wastewaters, drinking water, river water, and so forth. Worthwhile, BC has also been used for sequestering noxious gases, specifically CO_2_, CH_4_ and mitigating nitrogenous gas, i.e., N_2_O.^29^ Likewise, the utilization of BC has been further targeted to distinct soil components for various reasons inculcating pollutant removal, enhancing soil properties, and many more. The associated mechanisms for attaining these tendencies were predominantly due to physisorption and chemisorption mechanisms (complexation, precipitation, electrostatic interaction, ion exchange, capillary condensation, and sorption)^26^ occurring in the presence of BC. The availability of precise conditions for achieving appropriate wastewater treatment, mitigating gaseous pollutants, and remediating different soil types was better achieved by various influential parameters required for BC development, as they drive the BC properties and surface morphology. These parameters include the different types of feedstock material, production technique, temperature, and so forth*.*^30^^,^^31^

BC is a pyrolyzed material produced from carbonizing different feedstock materials at variable temperature ranges. The feedstock material used for the production of BC consists of inorganic and organic solid residue attained from living and non-living sources.^32^ The biomass used as feedstock material is distinctively classified into various types based on their coherent properties. Vassilev et al.^33^ divided BC into five classes which were aquatic biomass, woody waste, industrial waste, agricultural biomass, and human and animal waste. Likewise, Jafri et al.^34^ categorized biomass into two classes, i.e., woody (residue from forest and tree) and non-woody biomass (agricultural crop residue, industrial waste, and animal waste). Similarly, Hopkins et al.^35^ divided feedstock into lignocellulosic biomass and marine biomass. However, the BC developed from such feedstock material was highly affected by the persisting temperature and technique used.^15^ Till now, four different types of techniques are known to be involved in producing BC. The techniques consisted of hydrothermal carbonization, dry torrefaction, pyrolysis, and gasification which worked at a temperature range of 180°C–260°C, 200°C–300°C, 300°C–650°C and 600°C–1200°C respectively for producing BC from biomass.^36^^,^^37^^,^^38^^,^^39^^,^^40^^,^^41^^,^^42^ These production techniques and temperature influence the different surface morphologies of BC including specific surface area (SSA), porosity, and cation exchange capacity.^43^ For example, Li et al.^44^ mentioned that at greater temperature range, the porosity of BC increases. Likewise, increasing the temperature to about 400°C–800°C enhances the SSA and beyond that temperature negligible improvement of SSA occurs.^45^ Corresponding to temperature, feedstock material also affects the BC properties, where, higher water content and micro-organic molecule containing substances generate more porous BC.^46^ The BC derived from manures or biological solids contain lower SSA as compared to woody biomass due to occurrence of deformation, structural cracking and pore blocking.^15^ In contrast, these manures contain greater CEC and pH opposite to woody BC as oxidized surface and inorganic functional groups are generated upon BC surface during heating.^47^ Insoluble precipitates are formed during BC development creating sites for specific reaction and consequently facilitates the production of effective CEC on BC.^48^ These inherited BC properties with advanced porosity, SSA and CEC supports growth of distinct micro biota which favored efficient retention of nutrients and pollutants, enhanced soil properties and remediated several noxiuos gases.^15^^,^^49^ These active properties of BC with an ability to rehabilitate the prevailing environmental conditions (soil, air, and water) coherently make it a promising candidate to achieve various SDGs. The different goals of SDGs include various targets for uplifting the lives residing in the environment, whereby, the characteristics of BC depicted specific potential to help resolve partially or completely the targets of SDGs. So, further subsections will provide an in-depth analysis of the capacity of BC to achieve the different SDGs.

## Application of biochar for fulfilling sustainable development goals

**Figure 2:**
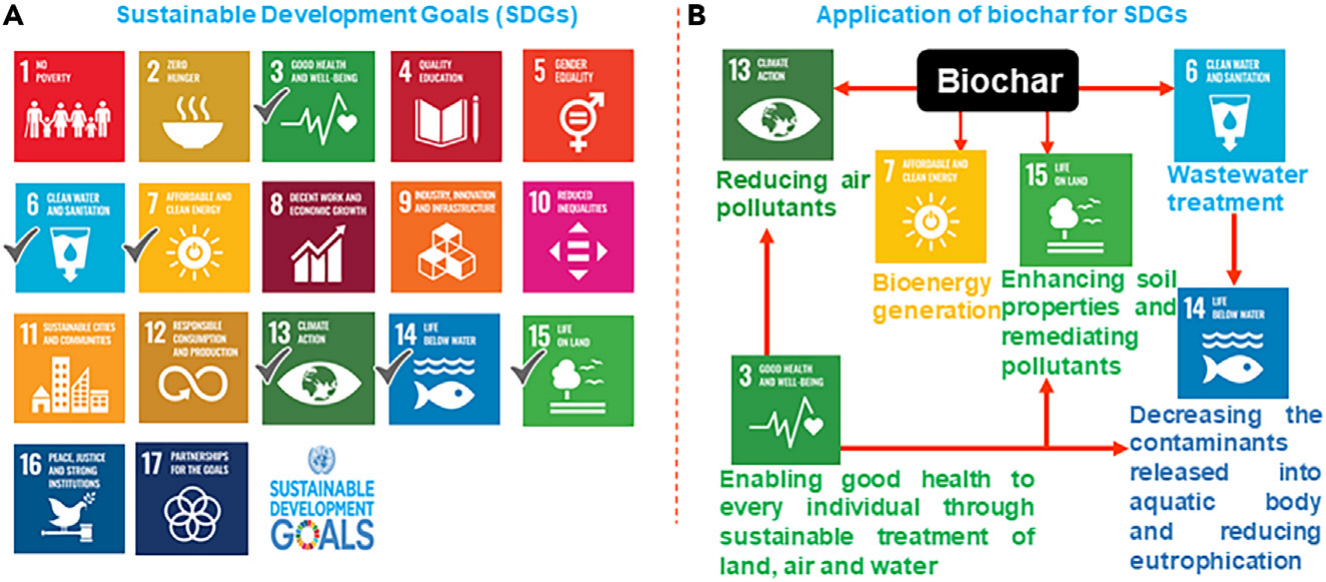
Schematic representation of total number of SDGs fulfilled by biochar (A) Total number of SDGs targeted to be achieved by 2030. Tick mark (✓) depicts the SDGs attained using biochar application; (B) Targeted SDGs achieved using biochar as a potential material.

Biochar possesses various essential inherent properties including sustainability, cost-effectiveness, high productivity, and so forth*.* that make it a viable material for extensively solving environmental pollution. As aforesaid, the influential physicochemical properties of BC have explicitly facilitated its application for remediating pollutants from various wastewater types, and soil environment, enhancing soil properties, reducing the release of toxic greenhouse gases, and sustainable energy generation in the environment. The utilization of BC for producing bioenergy and remediating, soil, water, and air resonates its applicability with the targets of SDGs. The validation of BC’s sustainable application has been confirmed strongly in the IPCC Report 2018, where, BC is regarded as a negative emission technology (NET).^50^ The properties and applicability of BC suitably fit to achieve some of the goals (provided in Figure 2) among the 17 SDGs which are targeted to be achieved by 2030. However, in the present review, it was estimated that BC could successfully afford to accomplish four SDGs. The tendency of BC to remediate a wide range of pollutants from wastewater is priorly targeted to SDG 6 depicting clean water and sanitation. Treating the water at source using BC and restricting the discharge of pollutants into open waterbodies would deliberately reduce the deleterious effects on various forms of life residing below water, and thus target to achieve SDG 14 (Life below water) simultaneously. Furthermore, using BC for soil productivity enhancement and contaminant removal renders the attainment of SDG 15 (Life on land). Similarly, using BC prohibits the emission of greenhouse gases into the environment apprehending the successful accomplishment of SDG 13 (Climate action). Meanwhile, the enhanced properties and cost-effectiveness of BC also contributed to effective bioenergy generation that supplemented the attainment of SDG 7 (Affordable and clean energy). Summarizing the overall potential of BC for alleviating environmental issues sustainably would persuade good health for each and every living individual residing on land as well as the aquatic body which is quite relatable to SDG 3 (Good health and well-being). So, looking into the vivid utilization of BC for acquiring the various SDGs, below provided subsections will depict an insight into the sustainable applications of BC to help improve the vital issues existing in different spheres of the environment.

### Reclamation of water (SDG 6: Improve clean water and sanitation)

Since past decades various physicochemical and biological treatment technologies have been actively designed to treat the enormous amount of wastewater being generated.^51^ Extensively used biological treatment methods include anaerobic digestion, activated sludge process, whereas, physicochemical treatment involves advance oxidation process (AOP), ultrasonic irradiation, and so forth*.*^52^ Interestingly, among these techniques, the process of adsorption has been of greater interest for remediating organic and inorganic wastewater pollutants.^53^ However, the primary application of adsorption was experimented using carbonaceous material such as activated carbon.^54^ Several experimental studies demonstrate pieces of evidence for applicability of low-cost and sustainable activated carbon for the treatment of organic and inorganic wastewater contaminants.^55^ In this regard, a rising trend had been introduced to prioritize and investigate carbonaceous materials for novel environmental remediation technologies. Consequently, the concept of BC has been introduced and affirmed by numerous research studies to deal with aquatic pollutants in a sustainable manner.^52^ Enormous experimental research confirmed BC as a potent adsorbent for treating inorganic pollutants such as heavy metals,^56^ conventional pollutants, and organic pollutants such as dyes,^57^ pesticides and many other contaminants^58^^,^^59^^,^^60^^,^^61^ from various aqueous solution. These efficient characteristics of BC also effectively serve the target of SDG 6 concerning “Clean Water and Sanitation.” As SDG 6 deals with sustainably generating clean water, the properties of BC affirm better reproducibility for achieving these targets. However, BC pertaining to SDG 6 has been discussed in detail in very limited studies. So, further subsections will provide an idea of BC and its global implications for treating pollutants from aqueous solutions and accomplishing SDG 6.

#### Heavy metals and other toxic elements

In recent years, the highly toxic nature of heavy metals has contributed to a greater surge in inculcating BC as an effective adsorbent for remediating these heavy metals. Application of BC has caused the efficient removal of a large number of heavy metals from several wastewaters originating from different sources. Due to the lower efficiency of conventional wastewater treatment systems,^3^ the use of BC has been widely accepted for treating these toxic metals from polluted wastewater. The efficiency and economic feasibility of BC induced greater efforts for exploring distinct BC types for enhanced removal of pollutants. Furthermore, for enhancing the performance of BC, different modification strategies have also been applied to increase the heavy metal adsorption process from aqueous solution. Such approaches led to the removal of a large number of heavy metals using BC that includes Cd, Cu, Ni, Zn, Pb, and so forth*.* As mentioned by Ni et al.*,*^62^ metal ions having a smaller ionic radius and greater electronegativity consisted of good binding affinity toward the surface functional groups, such as –OH, -COOH, of hard Lewis bases present on BC. As a result, surface complexation and cation exchange processes have a major contribution to the sorption of heavy metals on BC in a pH-controlled environment. Several studies have reported that the use of BC had reduced the concentration of heavy metals from wastewaters to a level lesser than the concerned drinking water standards including ion exchange with the available elements upon BC surface, i.e., Na^+^, K^+,^ and Ca^2+^; surface complexation reactions, electrostatic interaction; forming insoluble compounds through precipitation with ${SO}_{4}^{2-}$ and ${CO}_{3}^{2-}$; and physisorption.^56^ For example, Bandara et al.^63^ reported that the use of poultry litter BC removed about 99% of Cd(II) and Cu(II) from acid mine wastewater, where the initial concentrations were 0.58 and 2.28 mg/L respectively. According to WHO, the drinking level standard for Cu(II) was 2 mg/L, and the use of poultry litter BC efficiently treated the Cu(II) ions below the drinking standard level. The greater removal of Cu(II) as compared to Cd(II) ions was attributed to the presence of ${PO}_{4}^{3-}$ and ${CO}_{3}^{2-}$ ions present in BC that favored the occurrence of precipitation and surface adsorption and simultaneously enhanced the removal of Cu(II). Additionally, BC having higher N content possesses a higher affinity for Cu(II), thus, facilitating effective Cu(II) removal. Similarly, Manyuchi et al.^64^ reduced the concentration of heavy metals from gold tailing wastewater to a range below the WHO guidelines for effluent discharge. The study incorporated mixture of saw-dust and molasses BC for treating gold tailing wastewater, having initial concentration of 0.23 ± 0.01 mg/L of Cr^3+^, 0.80 ± 0.06 mg/L of Fe^2+^, 8.59 ± 8.60 mg/L of Zn^2+^, 0.92 ± 0.06 mg/L of Ni^2+^, 0.71 ± 0.04 mg/L of Pb^2+^, 0.12 ± 0.01 mg/L of Mn^2+^ and 5.53 ± 0.40 mg/L of Cu^2+^. It was observed that the BC was able to treat about 78.3%, 73.8%, 85.9%, 81.5%, 70.4%, 83.3%, and 90.8% of Cr^3+^, Fe^2+^, Zn^2+^, Ni^2+^, Pb^2+^, Mn^2+^ and Cu^2+^ respectively. The reduction in heavy metal concentration was attributed to the available retention time, pore size, and surface area of BC for adsorption. All the factors together uplifted the BC adsorption efficiency in treating heavy metals from the provided wastewater.

Various factors affect the removal of heavy metals from aqueous solution by using different types of biochar including, initial concentration of pollutants, pH of the solution, BC production temperature, adsorbent dosage, and so forth. For example, Cheng et al.^65^ used BC derived from poplar sawdust for treating Pb^2+^ and Cd^2+^ revealing a removal rate of 9.01% and 7.53% respectively at an initial concentration of 200 mg/L for each of the pollutants. Instead, when the initial concentration of Pb^2+^ and Cd^2+^ was 400 mg/L each, the removal rates were 6.95% and 9.81% respectively. The removal of Pb^2+^ and Cd^2+^ was due to the precipitation reaction with the mineral content of BC developed at 600°C. Also, the co-precipitation of Pb^2+^ and Cd^2+^ could also occur with metal ions such as K^+^, Mg^2+^, and Ca^2+^ due to surface complexation or electrostatic cation exchange. Pb^2+^ and Cd^2+^ were also possibly treated due to the interaction of heavy metals with π electrons in C=C. The cyclic aromatic π-system donates the π electron (as the cation- π binding site tends to be a weak bond) for interacting with Pb^2+^/Cd^2+^ and resultantly formed Pb-π and Cd- π.^66^^,^^67^

Moreover, differential results were observed with BC developed using different feedstock types (provided in Table 1). Earlier studies have reported the use of various types of BC involving agricultural waste, paper waste, marine waste, and so forth*.* for efficient heavy metal removal from different wastewater. The study reported by Singh et al.^68^ mentioned that using sugarcane BC provided a removal efficiency of 99.24%, 10.95%, and 95.52% for Cd, Fe, and Pb respectively. Instead using BC derived from plastics of polyethylene terephthalate and polyethylene demonstrated a removal rate of 28.31%, 4.0%, and 70.07% for Cd, Fe, and Pb, respectively. While, Das et al.^69^ utilized BC with different feedstock materials (maize stalk, *Lantana camara*, pine needle, black gram) for analyzing effective heavy metal removal from wastewater collected from the municipal treatment plant. The initial concentration of Cd, Cr, Pb, Zn and Cu were 0.19μg/L, 34.3μg/L, 1.67μg/L, 64.5μg/L and 51.2μg/L respectively and after treatment with different BC types, the removal rates were in the range of 52.6–94.7%, 83.1–88.1%, 94.6–77.8%, 90.1–94.5%, and 93.3–95.5%, respectively. The affinity of heavy metal ions differently to different BC types depicted that the removal rate depends greatly upon feedstock materials due to varying rates of surface electrostatic interaction, surface complexation, and precipitation mechanism taking place. Interestingly, when BC was modified with different forms of iron, the efficiency of BC material was enhanced. More pronouncing results were observed in modified BC due to the interaction of heavy metals with iron oxides and the simultaneous occurrence of an endothermic chemisorption mechanism. For example, Hasan et al.^70^ demonstrated that BC modified with nanoscale zerovalent iron could remove about 91.7% and 94.2% of Cd^2+^ and Zn^2+^ respectively, instead, unmodified BC could only treat about 42.6% and 42.2% of Cd^2+^ and Zn^2+^, respectively. Also, Liu et al.^71^ used BC alginate beads where BC was modified with ferric/ferrous sulfate. The results represented that on increasing the pH between 6 and 8, the adsorption capacity (Alginat BC: 25 mg Cd^2+^/g, modified alginate BC: 30 mg Cd^2+^/g) of both substances was increased. In addition, when these BCs were developed under different temperature conditions, the adsorption capacity of the adsorbent varied. The unmodified BC alginate beads and ferric/ferrous sulfate modified BC alginate beads were developed at 300°C, 500°C and 700°C, where, both the types of BC developed at higher pyrolytic temperatures (500°C and 700°C) depicted higher adsorption tendency for heavy metals. Likewise, when the temperature of the solution was increased from 24°C to 37°C in the same study, the adsorption capacity of the BC material was enhanced due to endothermic chemisorption reaction. Coherent results pertaining to the temperature modification of BC were observed by Singh et al.^68^ for treating Cd from an aqueous solution. Enhanced removal of Cd was provided by different feedstock material at a temperature range of 550°C as compared to BC developed at 450°C. BC with feedstock material as neem, sugarcane, polyvinyl chloride and polyethylene, polyethylene terephthalate, and polyethylene produced Cd removal efficiency of 45.86%, 98.36%, 7.89% and 3.97% at 450°C, whereas at 550°C, the removal rates were 66.40%, 99.24%, 24.58%, 28.31%, respectively. The reduced removal rates at lower temperatures were due to the production of BC with lesser surface area and higher non-carbonized organic matter available for adsorption which was opposite to the BC developed at higher temperatures. Oppositely, when Shan et al.^56^ compared the efficiency of BC developed at 350°C, 400°C, 500°C and 600°C, the results suggested that BC developed at lower pyrolysis temperature of 400°C showcased higher adsorption of heavy metals (Pb^2+^: 45.98 mg/g, Cd^2+^: 34.22 mg/g, Ni^2+^: 22.1 mg/g, Cu^2+^: 33.5 mg/g and Zn^2+^: 15.39 mg/g). The higher adsorption efficiency at this particular temperature was attributed to greater cation exchange and the existence of a more oxygen-functional group for productive adsorption.

**Table 1:** Applications of biochar for the treatment of various aquatic pollutants

Sl.no.	Type of biomass used	Production technique and temperature	Type of water	BC dosage	Efficiency (%)/adsorption capacity (mg/g)	Pollutant type	Initial conc.	Reference
1	Poplar saw dust	Heated at 600°C	River water	0.1g/100mL	9.01	Pb^2+^	200 mg/L	Cheng et al.^65^
					7.53	Cd^2+^	200 mg/L	
2	Hardwood	NA	Stream water	0.5g/50mL	99	Cu^2+^	0.015 mM/L	Richards et al.^72^
					100	Zn^2+^	0.015 mM/L	
3	Peanut shell	Heated at 600°C	Drinking water	14 g/L	86	Pb^2+^	2 mg/L	Pineda et al.^73^
	“Chonta” pulp			12 g/L	74	Pb^2+^	2 mg/L	
4	Corn cob			12 g/L	85	Pb^2+^	2 mg/L	
	Poultry litter	Pyrolysis at 550°C	Acid mine wastewater treatment	0.4g/40mL	>99	Cd(II)	0.58 mg/L	Bandara et al.^63^
					> 99	Cu (II)	2.28 mg/L	
5	Maize stalk, *Lantana camara*, pine needle, black gram	Pyrolysis at 600°C	Municipal treatment plant wastewater	2.5–5.0g/1L	78.5–86.5	As	16.3μg/L	Das et al.^69^
					52.6–94.7	Cd	0.19μg/L	
					83.1–88.1	Cr	34.3μg/L	
					94.6–77.8	Pb	1.67μg/L	
					90.1–94.5	Zn	64.5μg/L	
					93.3–95.5	Cu	51.2μg/L	
6	Pinewood	Pyrolysis at 600°C	Synthetic stormwater runoff	50mg/300mL	42.6	Cd	25.62 mg/L	Hasan et al.^70^
		Pyrolysis at 600°C + doped with nanoscale zerovalent iron			91.7	Cd		
7	Macro-algal bloom	Heated at 400°C–500°C	Synthetic wastewater (Solution pH 5)	0.250g/100mL	53.40	Cu	20 mg/L	Jaiswal et al.^74^
					27.25	Ni	10 mg/L	
					29.3	Co	10 mg/L	
					78.9	Cd	30 mg/L	
			Synthetic wastewater (Solution pH 2)		10.39	Cu	20 mg/L	
					7.65	Ni	10 mg/L	
					7.3	Co	10 mg/L	
					16.8	Cd	30 mg/L	
8	Jackfruit seed waste	Thermally activated with orthophosphoric acid at 500°C	Synthetic solution (pH 7)	0.1g	99.8	Fe (II)	5-100 mg/L	Khadem et al.^75^
					99.8	Cd (II)		
					99.6	Pb (II)		
					99.7	Cu (II)		
					61.6	Mn (II)		
9	Saw-dust and molasses	Ignited at 500°C	Gold tailing wastewater	50 mg/L	78.3	Cr^3+^	0.23 ± 0.01 mg/L	Manyuchi et al.^64^
					73.8	Fe^2+^	0.80 ± 0.06 mg/L	
					85.9	Zn^2+^	8.59 ± 8.60 mg/L	
					81.5	Ni^2+^	0.92 ± 0.06 mg/L	
					70.4	Pb^2+^	0.71 ± 0.04 mg/L	
					83.3	Mn^2+^	0.12 ± 0.01 mg/L	
					90.8	Cu^2+^	5.53 ± 0.40 mg/L	
10	Date seed	Pyrolyzed at 300°C	Synthetic aqueous solution	0.06g/50mL	90	As (III)	1 mg/L	Pal et al.^76^
	Delonix regia seed				87.7	As (III)		
11	(unmodified)	Pyrolyzed at 550°C–600°C	Synthetic aqueous solution	0.5g/50mL	14	Ni^2+^	100 mg/L	Sajjadi et al.^77^
	Pine wood (Ultrasonicated)				42	Ni^2+^		
12	Sugarcane	Pyrolyzed at 550°C	Synthetic aqueous solution	0.1–0.5g	99.24	Cd	20 mg/L	Singh et al.^68^
					10.95	Fe		
					95.52	Pb		
	Polyethylene terephthalate and Polyethylene		Synthetic aqueous solution		28.31	Cd	20 mg/L	
					4.0	Fe		
					70.07	Pb		
13	Acacia leucophloea	Pyrolyzed at 700°C	Synthetic solution	25-200mg/50mL	98	BR29	60 mg/L	Nirmaladevi et al.^57^
					98	RR2		
14	Cow dung	Pyrolyzed at 500°C	Synthetic solution	0.5–6.0g/100mL	97.0–99.0,	Methylene Blue	100 mg/L	Ahmad et al.^78^
	Rice husk				73.0–98.9			
	Sludge				71.0–99.0			
15	Aegle marmelos	Carbonized at 270°C	Synthetic solution	0.5–3.0g/100mL	74	Patent Blue	0.05 gm/L	Ahmad et al.^79^
16	Casuarina equisetifolia	Pyrolyzed at 500°C	Synthetic solution	5g/250mL	89.1	Methylene Blue	50 mg/L	Bharti et al.^80^
17	Crab shell	Pyrolyzed at 800°C	Synthetic solution	0.5 g/L	20	Malachite Green	4000 mg/L	Dai et al.^81^
18	Rice husk	Pyrolyzed at 400°C–600°C	Synthetic solution	1.5 g/L	96.5	Malachite Green	20 mg/L	Ganguly et al.^82^
19	Date Palm	Pyrolyzed at 400°C–600°C	Synthetic solution	0.1g/50mL	94.94	NO_3_	10 mg/L	Fseha et al.^83^
20	Rice husk (BC)	Pyrolyzed at 500°C	Synthetic solution	0.1g/30mL	10.12	NO_3_	10-40 mg/L	Sadeghi Afjeh et al.^84^
	Rice husk (BC + hydrogel)				34.3			
21	White oak+white cedar wood.	Pyrolyzed at 550°C	Synthetic solution	–	6.1–89.6	NO_3_-N	10 mg/L	Tian et al.^85^
	BC + zro-valent iron				30.6–95.7			
22	Steel pickling waste liquor and sugarcane bagasse (modified Fe/Ni)	Burned at 600°C	Synthetic solution	0.5g/50mL	98.39%	NO_3_	20-50 mg/L	Li et al.^86^
23	Rice straw	Pyrolyzed at 500°C	Synthetic solution	5 mg/50 mL	428.25 mg/g	U(VI)	50 mg/L	Ahmed et al.^87^
24	Pine needles	Pyrolyzed at 600°C	Synthetic solution	5 g/L	623.7 mg/g	U(VI)	11.9 mg/L	Philippou et al.^88^
25	Rice straw	Pyrolyzed at 500°C for 2 h	Synthetic solution	1 g/L	111.11 mg/g	F^−^	6 mg/L	Zhou et al.^89^
26	Rice husk	Pyrolyzed at 300°C and 700°C for 40 and 60 min	Synthetic solution	4 g/L	4.45 mg/g	F^−^	5 mg/L	Yadav and Jagadevan^90^
27	Tamarix hispida	Pyrolyzed at 350°C for 2 h	Synthetic and real wastewater	5 g/L	164.23 mg/g (synthetic wastewater), 86.69% (real wastewater)	F^−^	40 mg/L (synthetic wastewater), 3 mg/L spiked to 23 mg/L (real wastewater)	Habibi et al.^91^

The table depicts the reclamation of different types of water consisting of heavy metals, conventional pollutants, dye, and geogenic pollutants. The factors involving feedstock material, production technique, adsorbent dosage, and the type of pollutant affecting the removal mechanism are provided below.

Coherently, several studies have reported that providing a solution pH of around 4 to 6 facilitates effective heavy metal removal from various aqueous solutions. Jaiswal et al.^74^ mentioned that the heavy metal removal efficiency of BC gets enhanced when the pH of the solution is increased from 2.0 to 5.0. At pH 2, the BC could treat about 10.39%, 7.65%, 7.3%, and 16.8%, and at pH 5, the BC could remove about 53.40%, 27.25%, 29.3% 78.9% of Cu (II), Ni (II), Co (II) and Cd (II) respectively. At lower pH, the BC surface gets positively charged due to the existence of a higher concentration of H^+^ ions. As a result, the positively charged metal ions endure electrostatic repulsion from the protonated surface. Also, competition between ions of heavy metals and hydrogen atoms occurring for adsorption sites reduces the adsorption tendencies of the material.^92^^,^^93^ Oppositely, on increasing the solution pH, the surface of BC became negatively charged and the adsorption of heavy metals upon the BC surface was enhanced.^74^ Also, increasing the solution pH toward 6 produces stronger electronegativity upon the BC surface and strengthens the development of the bond between BC and heavy metal ions.^94^ Corresponding to this study, Khadem et al.^75^ reported that when BC was exposed to solutions with a pH range of 4–9, the BC represented higher removal efficiency at pH 7, where the removal rates were 99.8%, 99.8%, 99.6%, 99.7% and 61.6% for Fe (II), Cd (II), Pb (II), Cu (II), Mn (II) respectively, and the initial concentration of the heavy metal ions was 5–100 mg/L. A similar pH-dependent study was executed by Singh et al.^68^ which depicted a removal rate of 89.36%, 89.93%, 99.01%, 0.14%, 72.51%, and 98.78% for Cu, Pb, Cd, Fe, Ni, and Cr at pH 4; 91.60%, 81.88%, 95.52%, 99.24%, 99.03% and 4.81% for Cu, Ni, Pb, Cd, Cr, and Fe at pH 6; 34.03%, 72.10%, 58.64%, 61.07%, 49.08% for Cu, Ni, Pb, Cd, Cr and Fe at pH 8, respectively. Likewise, Shan et al.^56^ mentioned that among the provided pH range of solution between 2 and 6, BC produced effective adsorption at pH 5 for all the heavy metals (Pb^2+^: 46.7 mg/g, Cd^2+^: 28.3 mg/g, Ni^2+^: 18.75 mg/g, Cu^2+^: 35.05 mg/g and Zn^2+^: 12.65 mg/g due to greater presence of negative charge upon BC surface, hence, availing more scope for the adsorption of positively charged heavy metals.

Nevertheless, there are various other modulating factors that help enhance the BC tendency for effective wastewater treatment. Earlier studies have mentioned that the presence of organic compounds promoted the removal of heavy metal ions. Lin et al.^94^ utilized oxidized BC for the removal of heavy metals, where, the coexistence of benzoate improved the heavy metal treatment by chelating with the oxygen-containing group of BC and selectively caused the adsorption of heavy metals. Similarly, convincing results were reported by Singh et al.*,*^68^ where, increasing the BC dosage from 0.1 to 0.5g increased the removal efficiency of Cd, Pb, Cr, Fe, Ni, and Cu ions from 11.76 to 99.24%, 59.0 to 95.52% 87.62 to 99.36%, 0.60 to 10.95%, 11.56 to 43.31%, and 36.43 to 91.59%, respectively. Increasing the dosage rate of BC consequently increased the available sites for sorption and simultaneously the free sites upon the exposed surface area for the enhanced adsorption of heavy metals through different BC materials. Moreover, exposing the BC surface to different physical and chemical conditions changes the adsorbing properties and simultaneously the heavy metal removal rate of the adsorbent. For example, Lee et al.^95^ used three different types of BC derived from rice husk, wood chips, and their mixture for the adsorption of three heavy metals, i.e., Cd, Pb, and Zn, under varying conditions (acidic: HCl, alkaline: NaOH, oxidizing: H_2_O_2_, manganese oxide: KMnO_4_, iron oxide: FeCl_3_). The results reported that BC modified with KMNO_4_ had greater adsorption capacity due to an increased number of functional groups including -OH, C=O, and Mn-O. The enhanced removal was attributed to the increased cation exchange with Mn and surface complexation reactions using delocalized π electrons or –OH groups formed by the impregnation of MnO_x_. Furthermore, the enhancement of BC adsorption property was also carried out by ultrasonicating the desired BC, as reported by Sajjadi et al*.*^77^ The results revealed that unmodified BC was able to treat 14% of Ni^2+^, instead, ultrasonicated BC could remove 42% of Ni^2+^. The acoustic wave used for the modification of the BC material developed certain other porous structures upon the surface simultaneously improved the porosity of BC and facilitated effective adsorption for wastewater treatment. Moreover, in the same study, when unmodified and ultrasonicated BC were treated with 10%, 20%, and 30% of HNO_3_, around 4% increase in Ni^2+^ uptake in 10% and 20% HNO_3_ solution in comparison to ultrasonicated BC having 30% HNO_3_ concentration. Although, ultrasonication exfoliated the structure of BC, enhanced the pore surface area, and favored leaching of mineral content out of BC, still, increasing the HNO_3_ concentration to 30%, produced no improvement in Ni^2+^ uptake due to the entrapment of greater nitrate ion conc. in the microporous structure of BC.^96^^,^^97^

**Figure 3:**
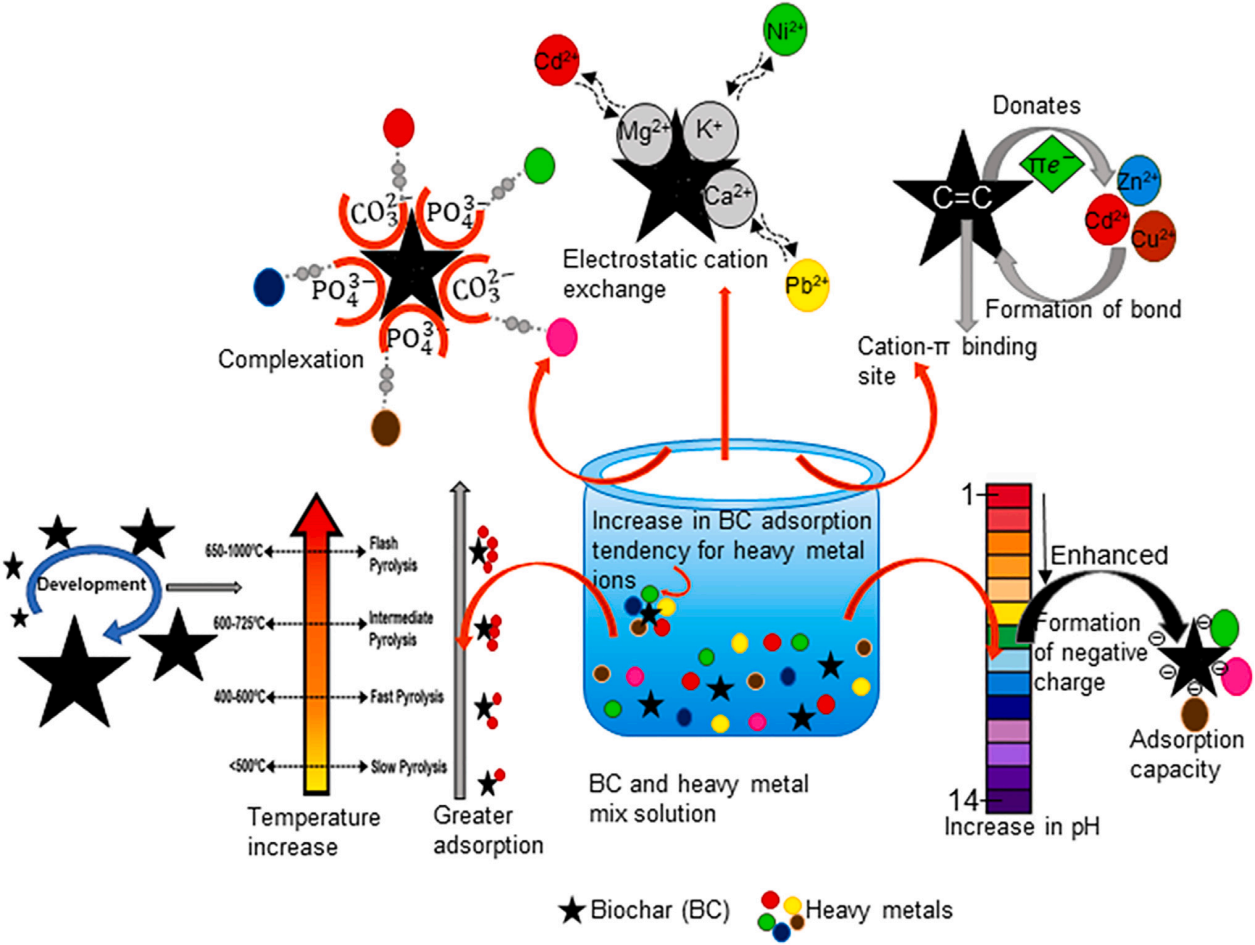
Mechanisms involved in the removal of heavy metals using biochar BC could sustain various mechanisms, i.e., surface complexation, electrostatic ion exchange, cation- π bonding, the role of pH, and temperature variation that favored effective removal of different toxic heavy metals from aqueous solutions.

The collective results from the different studies suggested that a higher rate of electrostatic attraction is produced between the different –OH and –COOH groups present on the BC surface and the heavy metal ions possessing greater electronegativity and smaller ionic radius. Likewise, the probable treatment of heavy metals using BC could also occur through the donation of π electrons from the weak cation- π binding site of BC (present in C=C) to the heavy metal (supposed to be X) and subsequently develops X- π heavy metal ions that possessed reduced toxicity. Such surface properties of BC adsorb the heavy metal ions through different phenomena (Figure 3) including surface complexation reactions, cation exchange, electrostatic interaction, physisorption, chemisorption, precipitation, and many more. These mechanisms are further affected by various controlling parameters such as retention time, pore size, BC surface characteristics, initial concentration of pollutants, pH of the solution, BC production temperature, modification of BC surface, and modification of solution through different oxidizing and reducing agents. Among these factors, BC surface characteristics tend to be the primary factor that should be taken into consideration for demonstrating effective heavy metal removal, as different materials possess a varying affinity to heavy metals due to modulations in the different influencing phenomena (such as precipitation, surface complexation, and so forth*.*) taking place. If these surfaces of BC were further modified with iron oxides, various endothermic chemisorption mechanisms come into existence that suitably removed heavy metal ions from the solution. However, the surface of BC also gets affected by different pyrolysis temperature that modifies its properties. BC produced at lower pyrolytic temperatures has a lower surface area and more non-carbonized organic matter opposite to that of BC at a higher pyrolytic temperature which reduces the affinity of heavy metals to BC. Likewise, the solution pH also affects the BC surface characteristics, where lower pH donates positive ions upon the BC surface and higher pH converts it into a negatively charged particle. At lower pH, H^+^ ions surmount the BC surface, thus causing electrostatic repulsion to the similarly charged heavy metal ions, while on increasing pH the BC surface gets more electronegative and attracts the toxic heavy metal ions. Similarly, various modification strategies and approaches have been applied to enhance the heavy metal remediation process using BC. Greater efforts are still required to attain a better understanding of the underlying mechanism for heavy metal removal. Although numerous adsorption studies are performed, comparatively, desorption studies are quite reluctant. A greater need for desorption study arises to study the sequential process taking place post adsorption. Additionally, toxicity tests need to be apprehended further to get a in-depth knowledge of BC sustainability post adsorption. Conclusively, the collected research studies demonstrate BC as a sustainable novel material for removing heavy metals from wastewater productively.

#### Geogenic pollutants

BC has proven to be a low-cost adsorbent for the removal of U (VI), F^−,^ and As (III) as well from different aqueous mediums. Such an approach has also been provided by Thakur et al.*,*^98^ where a detailed discussion related to recent studies on the use of BC for the removal of geogenic contaminants (U (VI), F^−^ and As (III)) from aqueous solutions have been mentioned. When individual research articles with respect to these contaminants have been considered, different experimental studies suggested multiple tendencies of BC for treating these geogenic pollutants. However, most of the studies had performed modifications of BC to enhance the removal rate of the geogenic pollutants. For example, Sen et al.^99^ reported that rice husk-derived BC modified with ferric oxide nanoparticles removed about 99.8% U (VI) from water at an initial concentration of 3 mg/L. Similarly, Li et al.^100^ synthesized magnetic BC which reduced the U (VI) to U (IV) with a removal efficiency of 90% at pH 4 and an initial concentration of 10 mg/L. Likewise, Ahmed et al.^101^ examined the HNO_3_ oxidized rice straw BC for the adsorption of U (VI) from contaminated water. They observed that the maximum sorption capacity of BC was 242.65 mg/g at pH 5.5 with an initial concentration of 50 mg/L. Oxidized BC showed better removal efficiency due to increased surface area, functional groups, and porosity. The major mechanism responsible for the adsorption of U (VI) was inner sphere complexation. Further, Zhang et al.^102^ investigated the sorption potential of pine needles BC for the removal of U (VI) from wastewater. They reported that BC removed about 99.9% U (VI) from industrial wastewater, where the initial concentration was 15 mg/L. Coherently, Kumar et al.^103^ used hydrothermally produced BC derived from switchgrass for the treatment of U (VI) contaminated water. They found that BC successfully removed the U (VI) with a maximum sorption capacity of 4 mg/g. Moreover, Ahmed et al.^87^ evaluated BC derived from rice straw modified with hydroxyapatite nanocomposite for the adsorption of U(VI) from an aqueous solution. Primarily, hydroxyapaptite is a calcium phosphate substance that has a high capability to remove pollutants from the environment. The adsorption potential of fabricated biochar was found to be 428.25 mg/kg whereas raw BC had an adsorption capacity of 110.56 mg/kg at an initial concentration of 50 mg/L and pH 5.5.^104^ Similarly, Philippou et al.^88^ removed U(VI) from water using magnetic BC from pine needles. Magnetic modification escalated the sorption capacity of BC through the incorporation of oxygenated functional groups on the BC surface. Magnetized BC was effective even after four sorption/desorption cycles.

Moreover, several studies have been carried out for the removal of F^−^ using BC. For example, Mohan et al.^105^ utilized pine wood and bark-based BC for the adsorption of F^−^ and exhibited maximum sorption potential of 7.66 mg/g and 9.77 mg/g respectively at pH 2. Similarly, Oh et al.^106^ removed F^−^ using BC prepared from orange peel and sludge at different pyrolytic temperatures. They revealed that F^−^ adsorption is a pH-dependent phenomenon. Maximum F^−^ sorption took place in acidic pH due to the protonation of functional groups resulting in electrostatic interaction. However, Goswami et al.^107^ used nanoscale rice husk BC for defluoridation and showed a maximum sorption potential of 17.3 mg/g at pH 7. The study reported the F^−^ sorption at neutral pH which is quite challenging. Likewise, Sadhu et al.^108^ evaluated watermelon rind BC for F^−^ uptake from groundwater and industrial wastewater. They reported a maximum sorption capacity of 9.5 mg/g at pH 1 and an initial concentration of 50 mg L^−1^. Further, Zhou et al.^89^ studied the rice straw-based BC loaded with metal oxide (La/Fe/Al) to remove the fluoride (F^−^) from water. Modification with tri-metallic oxide improved the efficiency of the BC, with a sorption potential of 10.85 mg/g for raw BC whereas modified BC showed F^−^ sorption capacity of 111.11 mg/g at an initial concentration of 6 mg/L. Modification improved the surface properties by introducing the hydroxyl groups on the BC surface and high adsorption capacity because La/Fe/Al oxides have a positive charge while fluoride has a negative charge The possible removal mechanisms were ion exchange and electrostatic interactions. Similarly, Habibi et al.^91^ employed LaCl_3_-activated BC derived from Tamarix hispida for the defluoridation of simulated and real wastewater. The BC exhibited an F^−^ adsorption capacity of 164.23 mg/g for simulated water and 86.69% for real wastewater. The prepared BC demonstrated high reusability, even after six cycles of reuse it showed an F^−^ removal efficiency of 53%. Co-existing ions such as bicarbonate, chloride, sulfate, and nitrate had only little effect on the F^−^ sorption. A combination of electrostatic attraction and precipitation was identified as the major mechanism for the removal of F^−^ from aqueous mediums.

Similarly, when the treatment of As (III) was considered, a variable rate of removal was observed under different influential factors. For example, Pal et al.^76^ compared the efficiency of Date seed (DS) and Delonix regia seed (DRS) by varying the initial concentration of As(III) from 0.2 to 2.5 mg/L. Upon increasing the initial concentration from 0.2 to 2.0 mg/L, the removal rate of As(III) increased from 92.75 to 95% and 91 to 93.7% in DS and DRS respectively. Increasing the heavy metal concentration acted as a driving force and surmounted the barrier of mass transfer to the surface of BC, thus, effectively adsorbing the pollutant. In contrast, increasing the initial concentration from 2.0 to 2.5 mg/L decreased the pollutant removal tendency which was consequent due to the saturation of the adsorption site upon the BC surface with an increase in the concentration of metal ions.^109^^,^^110^ The saturation of active binding sites upon BC reduced the vacant position for heavy metals to attach and then reduced the removal efficiency.^111^^,^^112^ As surface electrostatic interaction, complexation, and precipitation mechanisms occur at varying rates on different BC types indicating that the removal rate is highly dependent on the feedstock type. Corresponding results were reported by Pal et al.*,*^76^ where the removal efficiency of BC from DS and DRS provided distinct results for treating As(III) from an aqueous solution. Date seed BC represented a removal efficiency of 90.0% and BC derived from Delonix regia seed provided a removal efficiency of 87.7%. Moreover, previous studies have demonstrated that the presence of organic compounds facilitated the adsorption of heavy metal ions. Lin et al.^94^ employed oxidized BC for the sorption of heavy metals, where the presence of benzoate enhanced the sorption potential of the BC by chelating with the oxygenated functional groups present on the BC surface. On the other hand, when the dosage of adsorbent was uplifted, enhanced heavy metal removal rates were observed, as provided by Pal et al.*,*^76^ where increasing the dosage of BC materials from 40 to 60 mg enhanced the removal rate of As(III) from 84.6 to 86.5 to 87–90%. While, Das et al.^69^ utilized BC with different feedstock materials (maize stalk, *Lantana camara*, pine needle, black gram) for analyzing effective heavy metal removal from wastewater collected from the municipal treatment plant. The initial concentration of As was 16.3μg/L and after treatment with different BC types, the removal rates were in the range of 78.5–86.5%. As aforementioned, Pal et al.^76^ compared the effectiveness of BC derived from DS and DRS, where a similar trend for As(III) removal was observed with increasing pH. At pH 3, the removal rate of DS and DRS for As(III) was 90% and 87.4%, instead, at pH 5, the removal rates were 93.2% and 92.4% respectively. The improved removal rate at the specific pH was a result of the nullification of OH^−^ ions by the H^+^ ions upon the BC surface and reducing the impedance that prohibits the As(III) ion transport.

BC has been an effective adsorbent for the removal of geogenic pollutants such as U, F -, and As from water. From the available literature, it was observed that the fabrication of BC yielded better results. Effective modifications were acidic conditions, adding magnetic properties, and altering with metal oxides/hydroxides. The BCs showed high reusability, as they were effective even after five to six sorption-desorption cycles. Various feedstock were utilized for the production of BC, where crop residues such as rice husk, rice straw, maize stalk, softwood such as pine needles, and grasses were among the major feedstock materials. Moreover, the prior mechanisms responsible for the effective removal of these contaminants were electrostatic interactions, ion exchange, and inner sphere complexation.

#### Dye

Several research interventions have been made to analyze the capacity of BC for treating different organic pollutants including dyes, pesticides, phenols, and so forth. Among these, dyes occupy a major class of compounds having higher production rates due to their extensive utilization.^113^ The recalcitrant nature of these compounds poses a serious threat to nature,^12^ as a result, efficient treatment of these pollutants is highly essential. Consequently, different research interventions has demonstrated that the mechanism of adsorption plays a vital role in the detoxification and degradation of dyes.^12^ Earlier studies have reported BC acts as a better adsorbent for remediating several dyes from wastewater. The adsorption studies concerning the removal of dye using BC as an adsorbent displayed efficient removal of various anionic and cationic dyes from different types of aqueous solutions. Previous studies have reported that incorporating BC will suitably treat several types of dye molecules by exhibiting π-π interaction between the graphene layer of BC and the dye particle (this phenomenon becomes even stronger in the presence of OH^−^ groups upon the surface of BC), electrostatic attraction/repulsion and intermolecular hydrogen bonding between the dye molecule and the BC. Those mechanisms were likely affirmed by the experimental findings of Qiu et al.^114^ depicting the higher adsorption efficiency of rice straw BC with respect to activated carbon for eliminating Reactive Brilliant Blue and Rhodamine B dye. The FTIR results evidenced that the intermolecular hydrogen bonding (O-H----O) took place between the O atom of C=O present on BC and the OH molecule of dye. Consequently, the formation of electrical attraction between the CO and OH readily favored the adsorption of dye onto BC. Additionally, the π-π dispersive interaction between the graphene layer of BC and the aromatic rings of dye molecules also takes place.^115^ These π-π interactions are greatly influenced by the surface functional groups of BC, as the existence of lactone and carboxyl groups exhibit electron-withdraw nature and diminish the π-π bond by declining the π-electron density on BC surface. On the contrary, the prevalence of phenolic hydroxyls, which are electron-donating in nature, tend to strengthen the π-π interactions. So, the presence of the higher amount of these surface functional groups, i.e., phenolic hydroxyls, than lactones and carboxyls support π-π interactions and selective adsorption of dye. Corresponding results were reported by various other studies detailing the treatment of differently charged dye using BC. Nirmaladevi et al.^57^ studied removal of cationic dye (Basic Red 29: BR29) and anionic dye (Reactive Red 2: RR2) using *Acacia leucophloea* BC under varied environmental conditions (pH of solution: 2–10, mixing speed: 30–170 rpm, initial concentration of dye: 40–100 mg/L, contact time: 100 min, temperature applied: 320, 310 and 300 K, adsorbent dosage: 25-20mg). The study revealed an effective removal of 98% for both RR2 and BR29 at pH 2 and 10 respectively. Apart from the pH differences, other optimum conditions for removal of both dye remained equivalent, i.e., mixing speed of 170 rpm, 300K temperature, adsorbent dosage of 50mg, and initial dye concentration of 60 mg/L). A better explanation of such behavioral capacities of cationic and anionic dye was provided by Deniz et al.^116^ through the electrokinetic study of the adsorbing material. The positive charge on BR29 and the negative charge of RR2 posed a decreasing removal rate with pH reduction and pH enhancement respectively. These reductions of removal rate were associated with the repulsion of similar charges, i.e., positive charge (H_3_O^+^) on cationic dye due to the presence of excessive H^+^ ions at reducing pH and negative charge on anionic dye due to excessive OH^−^ ions at increasing pH. Moreover, pH_zpc_ (point of zero charge) of the adsorbing material also describes the adsorption nature of different types of dye. The existence of electrostatic attraction comes into play when the pH of the solution is greater than pH_zpc_, the adsorbing material develops a negative charge and favors the removal of cationic BR29. On the other hand, when the pH of the solution is less than pH_zpc_, the adsorbing material becomes positively charged and removes anionic RR2. The results were consistent with the findings of Roy et al.*,*^79^ where, the removal of patent blue dye using bael shell (*Aegle marmelos*) BC at different pH involving 2.7, 4, 6, 7, 8, 9.2 ad 10.4 were associated with a removal efficiency of 74%, 62%, 58%, 55%, 47%, 48%, and 39%, respectively. Moreover, there exist distinct influential parameters affecting the treatment of several dye types which include the type of feedstock material, pH of the solution, adsorbent dosage rate, initial concentration of dye, and many more. For example, Ganguly et al.^82^ experimented with the removal of Malachite Green dye using BC at a pH range of 2–8. The dye removal efficiency achieved by BC at pH 2 was 81.1% and was gradually enhanced up to 96.5% at pH 4, instead, on the arrival of an alkaline pH of 8, the dye removal efficiency was significantly reduced to 73%.

Furthermore, to demonstrate the effect of feedstock material for the treatment of dyes, Ahmad et al.^78^ compared the differential removal of cationic dye methylene blue from three different feedstock materials consisting of rice husk, sludge sample, and cow dung. It was observed that cow dung BC had the highest removal rate of 99%, instead, rice husk and sludge BC had approximately ∼99% removal of dye at a pH range of 7.0–9.0. As aforementioned, the electrostatic affinity between BC and the dye molecule formed due to the surface charge density of the adsorbent and the electronegativity of the dye molecule facilitated an effective dye removal mechanism. On the other hand, using BC derived from *Casuarina equisetifolia* seeds and immobilized with cells of Alcaligenes faecalis opposite to free cells of the same species for the treatment of methylene blue dye resulted in the treatment of 81.5% of the dye in the presence of free microbial cells and 89.1% removal in presence of BC immobilized with the microbial cells.^80^ The dye removal capacity was induced in the presence of immobilized cells by the synergistic effect of BC and microbial cells. The greater SSA of the *Casuarina* BC inculcated more active sites and pore space upon the BC surface that exhibited higher adsorption of the dye and larger target reaction sites for the microbial attack. Additionally, the biodegradation of the dye molecule within the pores of BC reformed the structural appearance of pores by modifying the dye fraction into renewed benign products that diffused out of the pore.^117^ Coherent results were reported by Dai et al.*,*^81^ where the greater SSA of crab shell BC removed about 20% of 4000 mg/L of malachite green dye from the aqueous solution.

Convincingly, a similar effect of contact time between the BC material and the dye molecule also facilitated the efficient treatment of toxic dye. As mentioned earlier, the study reported by Nirmaladevi et al.^57^ estimated the removal of differently charged dye through *Acacia leucophloea* BC by varying the contact time between 0 and 100 min, where, the cationic dye BR29 and anionic dye RR2 was removed within 10 min and 70 min respectively. These behavioral tendencies to different dye molecules were due to the saturation of the active reaction site that attained equilibrium conditions within this contact time. Likewise, the experimental results of Ahmad et al.^78^ suggested that over the 0–96 h contact time of methylene blue dye with each of three different BC materials produced removal efficiencies ranging between 69.8 and 94.8% for a period of 12–24 h and the highest removal rate was observed at 60–72 h having a removal rate of 91.3–99.3%. Depending on the net charge available upon the BC surface, the adsorption capacities of BC initially increase followed by stabilization, and further depict gradual reduction till reaching an equilibrium stage.

The overall studies suggested that the properties of BC actively enhance the removal rate of dye molecule through π-π interaction, intermolecular hydrogen bonding, and electrostatic attraction. The π-π interaction specifically occurs between the graphene layer of BC material and the aromatic ring of the dye molecule. The presence of electron-donating groups on the BC surface fastens the interaction, while, electron-withdrawing groups weaken the π-π interaction. However, when intermolecular hydrogen bonding is considered, the binding of BC with the dye molecule initiates with O-H-----O bonding, where the O molecule belongs to C=O of the BC material and the OH molecules were donated by the dye fraction for forming CO and OH interactions, thus alleviating the concentration of dye molecule from the aqueous medium. Moreover, the developed surface charge density upon the BC surface and the electronegativity of the dye lead to the formation of electrostatic attraction between the BC surface and the dye molecule. The generated electrostatic affinity was driven by the pH of the solution through various ways. At lower solution pH, prevalence of H^+^ ions on BC surface form repulsive forces to the positively charged cationic dye (consisting of H_3_O^+^), while attractive forces were formed for negatively charged anionic dye (OH^−^) and vice versa. Further, having a pH of solution greater than pH_zpc_ develops negative charge upon the adsorbent and thus attracts the positively charged cationic dye and vice versa. So, variation in pH results in the development of electrostatic attraction between the BC material and the dye particle, which simultaneously enhances the removal of dye from different aqueous mediums. Such versatility and flexibility make BC an ideal material to be used for the treatment of various colorants from wastewater. Although, in the present study much focus is given to the factors affecting the BC material for dye treatment. Still, more focused research objectives concerning the underlying kinetics and adsorption isotherm could be summarized to obtain a clear idea of the mechanistic approach and pathway followed by different BC materials for dye treatment from wastewater.

#### Conventional pollutants

In past decades, several traditional and advanced treatment technologies have been applied to remove conventional pollutants (phosphate, nitrate, ammonium) from wastewater ^3^ However, these technologies emerged with greater maintenance and operation costs with huge amounts of waste generation.^118^ Instead, when sorption-based methods using agricultural biomass were applied, the technology evolved to be user-friendly, cost-effective, and highly efficient for conventional pollutants. Following the sorption methods for conventional contaminants, when BC was used as an adsorbent, the experimental findings demonstrated highly effective results for such contaminants.^119^ To confirm such potential of BC, Fseha et al.^83^ incorporated date palm BC for removing NO_3_ from an aqueous solution. The results revealed that BC was able to treat about 86.52% of NO_3_ (initial concentration: 10 mg/L) at a BC dosage rate of 0.05 g/L and the efficiency further enhanced to 94.94% when the dosage was uplifted to 1 g/L. Such increased treatment efficiency was a result of higher active reaction sites available for the molecule at higher dosages to adsorb upon the BC surface. Likewise, when the pH of the solution was 4, the efficiency of the BC material was 86.21%. While, increasing the solution pH to 6, enhanced the efficiency of the system to 91.38%. However, improving the pH between 8 and 10 dropped the removal rate due to the competition of negatively charged NO_3_ ion with similar charge bearing hydroxide ions to adsorb upon the active sites of the BC surface. These efficient pollutant removal properties of BC also vary with vivid feedstock materials application. Sadeghi et al.^84^ compared the efficiency of three different types of material including BC, hydrogel and composite of hydrogel and BC for removing NO_3_ from aqueous solution. The results reported that the BC infused with hydrogel (34.3%) had the highest capacity for NO_3_ removal followed by standalone hydrogel (14.4%) and then by standalone BC (10.12%). Likewise, Adesemuyi et al.^120^ compared the BC derived from elephant grass at different pyrolysis temperatures. The BC derived at a higher temperature,600°C (96%) outperformed the one prepared at a lower temperature, 400°C (91%). Similarly, Sui et al.^121^ mentioned that the BC material had higher NO_3_ removal efficiency (97.0 ± 1.1%) at the production temperature of 600°C, whereas the below (400°C: 84.2 ± 2.6%) and above (800°C–1100°C: 28.0 ± 1.7%) that temperature range reduced efficiency of NO_3_ removal was observed. The study reported that adding BC with a production temperature of 600°C produced 3.5-, 2.6-, 1.1- and 1.2- times greater removal of NO_3_ in comparison to BC developed at a temperature range of 1100°C, 1000°C, 800°C and 400°C respectively. The capacity to treat NO_3_ effectively using BC produced at higher temperatures was accompanied by greater porosity through uplifted levels of π-π interaction or van der Waals’ interaction.^120^

Moreover, the physiological properties of BC and its tendency to release several nutrients, organic N and organic C could adhere to various types of denitrifying microbial consortium upon its surface for selective nitrate adsorption.^122^^,^^123^ The BC has greater synergy with denitrifiers, especially *Pseudomonas* N3, that favored up-regulation of NAR (nitrate reductase) and the *narG* gene expression associated with stimulated electron transport system activity (ETSA) for improved nitrate removal. Such influential effects of physiological properties (SSA and porosity) induced due to exposure of biomass at higher temperatures, solution pH, increasing the contact time, and so forth*.* were reported by various other studies for apprehending positive nitrate removal.^124^ As a result, to assess the activity of BC with denitrifiers, Bock et al.^123^ mentioned that denitrifying bioreactors consisting of BC and fed with 35 mg/L of initial NO_3_ concentration could remove about 86% of NO_3_ within 18h and 97% within 72h. On the contrary, control reactors in the absence of BC treated only 13% of NO_3_ at 18h and 75% at 72h. The higher SSA and the porous surface of BC provided an appropriate surface for retaining oppositely charged ions, water, and other organic compounds favoring the growth of denitrifying bacteria. These microbial communities further supplemented the denitrification process for assisting enhanced NO_3_ removal in the presence of BC.^125^ The occurrence of these denitrification processes can even prevail in the presence of BC and zero-valent iron which treated about 30.6–95.7% of NO_3_-N in comparison to control that could treat about 6.1–89.6%.^85^ The improvement in treatment efficiency was due to BC's ability to retain water and produce anoxic environment by donating electrons and facilitate effective denitrification. This similar strategy was observed in BC modified with Fe/Ni (BC-Fe/Ni) for nitrate removal as reported by Li et al.*,*^86^ where BC-Fe/Ni could amend 98.39% and nanoscale Fe/Ni treated 93.55%, while nanoscale zero-valent iron could remove only 86.53% of NO_3_. In the presence of bimetallic ions upon BC, no other metal toxicity was observed due to the adsorption of metal ions upon BC surface and no chance occurrence of metal release into the surroundings. Simultaneously, the nitrate removed from the solution turns majorly into ammonium ions, instead, a minor concentration remains as nitrite ions. Thus, iron-modified BC has a greater capacity to amend NO_3_ rather than unmodified BC with reduced metal toxicity.

Sometimes, BC fostered similar behavior for treating PO_4_ ions from different aqueous solutions through precipitation, ligand exchange, adsorption, surface complexation reactions, and many more.^126^ Several studies have reported that the application of BC induced better PO_4_ removal at higher temperatures. For example, Ramola et al.^127^ compared the efficiency of rice-husk BC produced at three different temperature ranges, i.e., 300°C, 500°C, 700°C. The experimental results revealed that BC developed at the highest temperature of 700°C produced the highest PO_4_ removal rate of 81–87%, whereas the BC at lower temperature could remove as much as 21% PO_4_ from the aqueous solution. At a greater temperature range, chances for the release of CO_2_ from BC exist which consequently causes a secondary reaction between the emitted CO_2_ and the C content of the BC matrix. As a result, the closed pores become open and the porosity gets enhanced for possible adsorption to take place. Additionally, at higher temperatures the hydrophobicity of BC also decreases due to lower levels of O, H content, and functional groups with the simultaneous formation of water clusters upon the BC surface that induces effective PO_4_ adsorption.^128^^,^^129^^,^^130^ As a result, increasing the pyrolysis temperature modifies the physicochemical properties of BC, hence, increasing the surface area and porous nature of BC. This avails higher active adsorption sites for PO_4_ to attach. Moreover, studies have reported that the removal of PO_4_ by BC is a physical as well as chemical adsorption process. The FT-IR spectrum of BC depicting the presence of tautomeric aciform of nitrite, protonated amine, and alcoholic forms develops electrostatic interaction with the PO_4_ ions through physisorption. Additionally, the XRD pattern resulted in the presence of Ca and Mg ions that favored effective chemical adsorption of PO_4_.^131^ Similarly, Yao et al.^132^ acclaimed that the removal of PO_4_ from aqueous solution was a surface adsorption phenomenon that occurred due to the prevalence of MgO particles upon the BC surface. Metal oxides have a greater capacity for the adsorption of anions, such as phosphate. In the presence of water, the surface of metal oxides gets hydroxylated and then attracts either a negative or positive charge with respect to the pH of the solution. The existence of similar surface functional groups upon the BC surface favors the efficient adsorption of phosphate ions from the solution upon its surface.

The overall results suggested that the inherent properties of BC make it a suitable candidate for the effective treatment of various types of water. The porosity is the major reason for such nature of BC as it sustains various chemical and biological reactions to occur and potentially remediate the toxic pollutants. Meanwhile, the SSA of BC also primarily facilitates different surface complexation reactions, ion exchange mechanisms, and pore-filling processes to effectively adsorb the existing conventional and advanced pollutants. These surface properties of BC are driven by the feedstock type, pH of the solution, pyrolysis temperature, contact time, adsorbent dosage, type of modification upon BC surface, and many more. Such factors upon modifying the surface properties of BC enforce the electron-enriching mechanisms (π-π interaction, hydrogen bonding) that support the binding or breakdown of the pollutant molecule. These favorable binding of pollutants upon BC surface navigates its further use for different applications. The use of spent BC post-treatment of heavy metals and geogenic pollutants has been used for developing electrodes and other bioenergy applications. Likewise, while treating the conventional pollutants, nitrogen, and phosphorus adsorbed BC will be further used as slow-release fertilizer. This could help improve the soil properties and qualities. These acquired characteristics of BC will favor the sufficient growth and nutritional value of plants. As a result, N and P adsorbed BC will contribute to a major portion of enhanced food security by improving soil properties and plant life. Based upon these characteristics of BC, further sections are designed to provide insights into the role of BC in reclaiming the contaminated soil and immobilizing the prevailing pollutants within the medium.

### Soil amendments (SDG 15: Advance life on land)

The rapid increase in population, urbanization, and industrialization has reduced the agricultural areas and increased the demand for food. Consequently, the application of chemical fertilizers and pesticides has escalated, leading to the deterioration of soil health. While several techniques have been developed for soil amendment, there is still a significant demand for organic and sustainable remediation methods/technologies. To attain these needs, various research studies have suggested biochar and its related technologies as one such innovative, cost-effective, environmentally friendly, and sustainable soil amendment approach gaining rapid attention. As aforementioned, BC is a solid carbonaceous product derived from the thermal degradation of biomass that consists of high surface area, porosity, and oxygen-rich functional groups which makes it suitable for a wide range of applications involving the remediation of soil and water ecosystems.^133^^,^^134^^,^^135^ These properties of BC associated with better soil amendment characteristics make it an attractive tool to achieve the SDGs, specifically SDG 15, pertaining to advancing life on land.^50^ As improving the soil quality will favor the enhancement of the nutritional value of crops which when available for consumption will sufficiently provide enough nutrition to the consumer. So, utilizing BC stands as a viable option as it could adsorb nutrients better and is easily available and cost-effective in nature, hence, proven affordable to every individual. As a result, different experimental studies have been executed to explicitly study the role of BC in enhancing soil properties. Consequently, several research studies have reported BC as an effective material for soil remediation due to its ability to adsorb various contaminants.^13^^,^^136^ It enhances soil fertility, soil structure, electrical conductivity (EC), and cation exchange capacity (CEC), and aids in nutrient retention.^133^^,^^136^^,^^137^^,^^138^^,^^139^ So, further subsections will provide a detailed discussion of how BC can improve soil health and remediate various contaminants from the soil.

#### Utilization of biochar for contaminants immobilization in soil

BC has gained much attention in recent years for immobilizing various pollutants in soil (detailed in Table 2) through its high surface area, porosity, and functional groups (such as carbonyl, carboxyl, and hydroxyl) present on the surface. Studies have demonstrated that BC can effectively mitigate soil pollution by adsorbing organic contaminants such as polyaromatic hydrocarbons (PAHs), pesticides, dioxins and inorganic contaminants such as As, Pb, Cd, and Cu. For example, Kumar et al.^140^ investigated the wheat straw BC pyrolyzed at 500°C (WSB500) to remediate contaminated soil. WSB500 showed a maximum removal efficiency of 83.7% with an initial concentration of 8 ppm due to increased surface area, pH, porosity, and surface functional groups. In another study fungus-based BC derived from Trichoderma atroviride hyphae effectively removed the Cd from saline-alkali soil up to 95.10%.^141^ Likewise, Cu and Pb contaminated soil was amended with wheat straw and sludge composite BC, and the contamination content was reduced up to 36.81% for Cu and 25.61% for Pb, respectively.^142^ Khan et al.^143^ utilized BC from sugarcane bagasse and poplar wood to reduce Cr and Pb from mine-polluted soil. Maximum reduction was observed using sugarcane bagasse BC, with 50.4% for Cr and 59.4% for Pb, respectively. This was due to the higher soil pH and CEC of sugarcane bagasse BC than the poplar wood BC.

**Table 2:** Application of biochar for pollutant immobilization in soil microclimate

Type of biomass used	Production technique and temperature	Pollutant	Type of study	BC dosage	Initial conc.	Removal efficiency	Reference
Wheat straw	Pyrolysis at 500°C	As	Lab scale experiment	0.375 g in 5 g soil	8 mg/L	83.7%	Kumar and Bhattacharya^140^
Ferromagnetic bamboo- based BC	Pyrolysis at 500°C for 2 h	As	Lab scale experiment	5 g in 500 g soil	142.5–165 mg/kg		Zhang et al.^144^
Trichoderma atroviride hyphae (fungus)	Pyrolysis at 500°C	Cd		100 mg/kg		95.10%	Zhao et al.^141^
Wheat straw and sludge composite BC	Pyrolysis at 500°C	Cu, Pb		2.4 and 4 g in 80 g soil	421.9, 681.4 mg/kg		Wang et al.^142^
Sodium alginate based magnetic porous BC	Pyrolysis at 300°C	Cd, As	Pot study	0.5 g in each pot	1.55 ± 0.08, 1.26 ± 0.003 mg/kg	79.35%, 32.54%	Liu et al.^145^
Magnetized cedar sawdust	Pyrolysis at 300°C for 2 h	As, Pb, Cd	Incubation experiment	1:10 (w:w) biochar to soil ratio	47.3 ± 6.7, 729 ± 92, 12.1 ± 4.4 mg/kg	28%, 32%, 25%	Wan et al.^146^
Miscanthus straw pellets	Pyrolysis at 700°C for 12 h	Cd, Cu, Pb	Incubation experiment		25 mg/kg	65%, 39%, 16%	Achor et al.^147^
British Oak, Ash, Sycamore and Birch derived BC and green waste compost		Pb, Cu	Pot study		21,000, 600 mg/kg	29.4%, 44.5%	Karami et al.^148^
Broiler litter	Pyrolysis at 700°C for 1 h	Cu	Lab scale experiment	20% (g biochar/g soil)			Uchimiya et al.^149^
Oak wood	Pyrolysis at 400°C	Pb	Pot study	25 g in 500 g soil	4626.39 mg/kg		Almaroai et al.^150^
Poplar and conifer		Phenanthrene and pentachlorophenol	Lab scale experiment	20 and 50 mg/g	150 mg/kg	7.9%, 88.8%	Rao et al.^151^
Oak wood	Pyrolysis at 400°C	Pb	Lab scale experiment	60 g in 1.2 kg soil	11,100 mg/kg	75.8%	Ahmad et al.^152^
Dairy manure	Pyrolysis at 450°C for 4 h	Pb and Atrazine	Incubation experiment	12.5 and 25 g in 500 g soil	70,000 and 50 mg/kg	72-89%, 53–77%	Cao et al.^153^
Rice husk	Pyrolysis at 500°C for 90 min	Cu, Cr, Zn	Pot study	0.5%, 1%, and 2% (w:w)		65.6%, 96.9%, 42%	Wang et al.^154^
Rice straw	Pyrolysis at 600°C	Cd	Incubation experiment	5% (w:w)		26.4%	Xu et al.^155^
Bamboo	Pyrolysis at 400°C	Ni	Pot study	90 g in 3 kg soil	100 mg/kg	38,2%	Hannan et al.^156^
Tobacco stem	Pyrolysis at 450°C	Cd	Pot study	1% and 2% (w:w)	30 mg/kg	44.44%	Yao et al.^157^
Poplar wood (PW) and sugarcane bagasse (SB)	Pyrolysis at 550°C	Cr and Pb	Pot study	3% and 7%	23, 6 mg/kg	PW:23.2 and 29.3%, SB: 50.4, 59.4%	Khan et al.^143^
Corn residue	Pyrolysis at 500°C	Cd	Incubation experiment	2%	30 mg/kg	37.75%	Moradi and Karimi^158^
Olive pulp	Pyrolysis at 350°C	Ni	Pot study	150 g in 1 kg soil	50 mg/kg	60%	Turan et al.^159^

The table discusses the different types of pollutants immobilized in soil medium in the presence of various affecting parameters such as type of feedstock material, production technique, and temperature, type of pollutant, experimental conditions, and the concentration of pollutants.

Furthermore, several studies have mentioned that the modification of BC in different environmental conditions favored efficient pollutant removal from soil microclimate. For example, Zhang et al.^144^ treated the As contaminated soil with ferromanganese modified bamboo-based BC. The prepared BC not only remediated As but also improved the soil properties such as soil organic matter, CEC, NH_3_, N_2_, and available phosphorous due to the addition of Fe and Mn onto BC. The findings reported a complexation of pollutants with the oxygen-containing functional groups of BC as the major contaminant treatment mechanism. Similarly, Liu et al.^145^ utilized the alginate-based magnetic porous BC spheres to reduce the bioavailability of Cd and As in contaminated soil. They found that BC significantly reduced the concentrations of Cd by 79.35% and As by 32.45% in the soil through hydrogen bonding and electrostatic attraction between contaminants and the BC. Likewise, Wan et al.^146^ treated the multi-contaminated soil (As, Pb, and Cd) with magnetized cedar sawdust BC. In this study, the magnetic BC showed strong potential in the simultaneous removal of As, Pb, and Cd by 28%, 32%, and 25% respectively from the soil. Further, Karami et al.^148^ examined the synergistic effect of BC and green-waste compost to regulate the Cu and Pb mobility and their uptake in ryegrass vegetation. The amendments were mixed with contaminated soil and then their effects on plant uptake were measured. Compost amendment reduced high Pb concentrations in pore water, while biochar was more effective at reducing pore water Cu concentrations. The study showed that the two amendments had metal-specific suitability for treating contaminated soil. Similar to the above-mentioned study, Beesley et al.^160^ studied the potential of BC and green-waste compost to reduce the mobility, bioavailability, and toxicity of trace elements in multi-element contaminated soil. BC was found to be most effective in reducing Cu and As concentrations, as well as PAHs, indicating its potential for contaminated land remediation.

Further, Wang et al.^154^ utilized the BC-mineral (vermiculite-kaolin-attapulgite) composite for the immobilization of heavy metals (Cu, Cr, Cd, and Zn) from naturally contaminated soil. The composites increased the heavy metal removal rate due to increased surface area, pore volume, and pore diameter. The attapulgite-BC composite raised the soil pH from 3.77 to 5.18. The highest reduction was observed for the kaolin-BC composite, which was effective in reducing 96.9% Cr, and the attapulgite-BC composite reduced 65.6% Cu and 42% Zn, respectively. There was no significant reduction observed for Cd. Moreover, Uchimiya et al.^149^ used BC as a soil amendment to sequester Cu. They evaluated the sorption capacity of BC on two different soils: clay-rich, alkaline San Joaquin soil and eroded acidic Norfolk sandy loam soil. Results showed that San Joaquin soil showed better Cu sorption capacity than the Norfolk sandy loam soil through the cation exchange mechanism. However, Almaroai et al.^150^ examined the effect of BC on inhibiting Pb uptake to maize in saline water irrigated soil. It was found that BC increased the electrical conductivity (EC), water-soluble cations and anions, and decreased the water-soluble Pb concentration. Correspondingly, Rao et al.^151^ explored the poplar and conifer-derived BC for the remediation of POPs, phenanthrene, and pentachlorophenol from the soil. The remediation efficiency of conifer-based BC was better than poplar based due to its higher surface area of 114.67 m^2^/g and pore volume of 0.067 cm^3^/g than poplar-derived BC with surface area and pore volume of 76.88 m^2^/g and 0.047 cm^3^/g. The conifer-based BC showed a removal efficiency of 7.9% for phenanthrene and 88.8% for pentachlorophenol. Likewise, Cao et al.^153^ investigated the effectiveness of dairy-manure BC in immobilizing heavy metal Pb and organic pesticide atrazine in contaminated soils. BC prepared from dairy manure was found to be effective in immobilizing both atrazine and Pb, with its effectiveness increasing with incubation time and biochar rates. The study also found that biochar’s phosphorus reacts with soil Pb to form insoluble hydroxypyromorphite Pb_5_(PO_4_)_3_(OH), which was responsible for soil Pb immobilization. Likewise, Turan et al.^159^ investigated the olive pulp BC and calcite composite to remediate Ni-polluted soil. It was observed that amended soil showed increased soil pH by 1.16 units and showed a significant reduction of 60% compared to the control.

**Figure 4:**
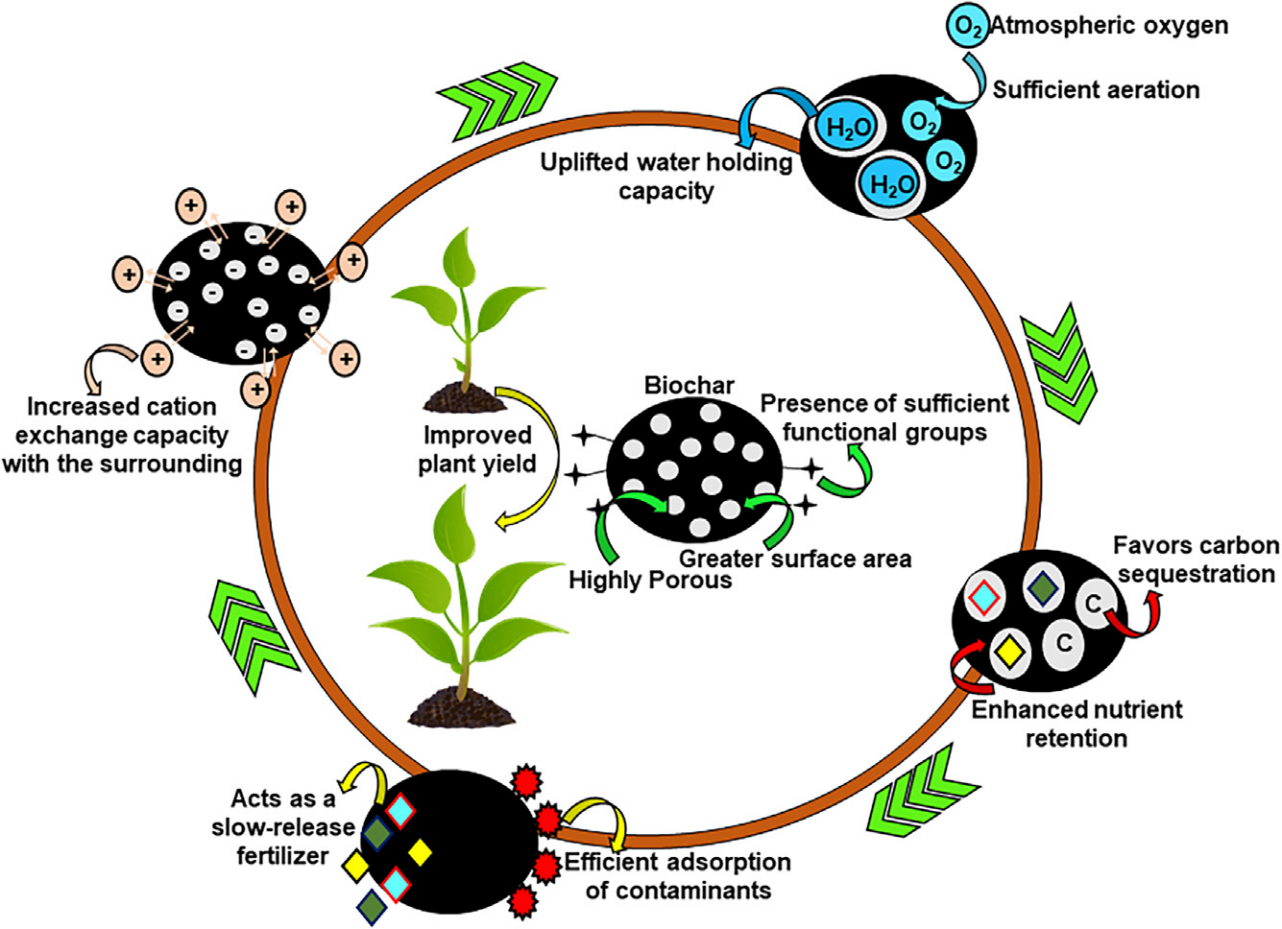
Mechanisms associated with biochar for efficient soil amendment and pollutant treatment The porous nature of BC comprises several intricate characteristics that make it a suitable material for contaminant immobilization and soil amendment studies. These characteristics include uplifted water holding capacity, enhanced nutrient retention tendency, efficient material for contaminant adsorption, slow-release fertilizer properties, and enhanced rate of cation exchange capacity with the surroundings. In addition, the highly porous nature, and greater surface area associated with the presence of sufficient functional groups support improving the soil quality, thus improving the plant yield in response.

Based on the collective results from different research interventions, the pollutant immobilization processes in soil microclimate through BC are provided in Figure 4. The results demonstrated that the primary focus for treating contaminated soils should be the preparation of pollutant-specific BC. Such BC material will uplift the rate of electrostatic attraction and hydrogen bonding that majorly comes into action during pollutant immobilization in soil. Also, developing pollutant-specific BC will improve the influential properties such as increasing the surface area, porosity, and surface functional groups of the developed material. These characteristics of BC also get increased by surface modification with different materials or aligned with different mineral composites. As a result, the CEC of the BC material gets enhanced and fastens the pollutant exchange process between the soil environment and the BC. Thus, BC facilitates the effective immobilization of various toxic pollutants from the soil environment upon its surface. So, such pollutant removal studies related to BC demonstrate it as a highly essential material that could boost soil quality by the primary treatment of various organic and inorganic pollutants. Still, an in-depth analysis of the involved mechanisms in the microclimate of soil for such processes is greatly required. So, the further section will discuss the associated soil parameters enhanced through the application of BC.

#### Enhancement of soil properties

It is widely known that the application of BC to soil improved its properties (detailed in Table 3), and crop yield, and removed toxic pollutants from the soil. BC has increased soil fertility and enhanced soil health by increasing soil pH, CEC, water-holding capacity, carbon sequestration, microbial diversity, and nutrient retention.^161^^,^^162^ Additionally, BC increases soil nitrogen retention by reducing gaseous loss and leaching. It also enhances the soil phosphorus availability by slowing down the leaching process.^163^ A meta-analysis on the influence of BC on the physicochemical properties of soil and crop productivity was conducted by Singh et al*.*^162^ The findings suggested that soil pH affects soil quality and fertility. It was also observed that BC improved the CEC, pH, and soil organic carbon by 20%, 46%, and 27%, respectively. In addition, the study evaluated the effect of pyrolytic temperature on BC properties and suggested that higher pyrolytic temperatures increased the soil porosity and reduced the bulk density while lower pyrolytic temperatures increased the microbial diversity. Likewise, Abrishamkesh et al.^164^ evaluated the impact of BC on alkaline soil and lentil growth. The study mentioned that BC enhanced the CEC, organic carbon, and available K, and reduced the bulk density, thus, simultaneously ameliorating the soil quality and plant growth. Similarly, when Savannah wood-based BC was incorporated into the soil for the enhancement of maize crop productivity, BC increased the crop yield better in sandy loam soil than silty loam soil indicating the effectiveness of BC on different soil textures.^165^ Further, Montagnoli et al.^166^ reported that BC-treated soil promoted the soil pH, nutrient availability, moisture content, and root and canopy development. Moreover, Abujabhah et al.^167^ explored the effect of BC in different soil types (brown sandy loam, black clay loam, and non-reactive red loam) with different loading rates of BC. It was observed that an increase in the loading rate of BC improved the soil conditions by raising soil pH, moisture content, and carbon levels.

**Table 3:** Application of biochar for soil amendment

Type of biomass used	Production technique and temperature	Type of soil	Biochar dose	Experimental condition	Remarks	Reference
Sewage sludge	Pyrolysis at 500°C for 6 h			Pot study	58–63.2% reduction in bioaccumulation of PAHs with respect to the control.	Khan et al.^168^
Hardwood derived BC and green waste compost				Pot study	>50% PAH concentration reduced, and decrease in mobility, bioavailability, and toxicity of trace elements.	Beesley et al.^160^
Rice husk	Pyrolysis at 250°C–300°C and 450°C–500°C	Alkaline	0.4, 0.8, 1.6, 2.4, and 3.3% by weight	Pot study	Enhanced CEC, organic carbon, available K, and reduced bulk density.	Abrishamkesh et al.^164^
Savannah wood	Pyrolysis in hot tail oven	Sandy loam and silt loam	3 t/ha	Pot study	Enhanced the maize crop productivity	Yeboah et al.^165^
Orchard pruning biomass	Pyrolysis at 500°C for 3 h	Alisol		Rhizobox-flatbed scanner system	Increase in soil pH by 1.3 units, nutrient availability, moisture content, root and canopy development	Montagnoli et al.^166^
Eucalyptus green waste	Gasification at 650°C–750°C	Black clay loam (BCL), red loam (RL), brown sandy loam (BSL)	32.5, 65, and 130 g in 1300 g soil	Pot study	improved soil pH from 4.5 to 4.83 in BSL, from 4.6 to 4.98 in RL, increased the moisture content by 10% in RL, 22% in BSL, 10% in the BCL, and carbon levels.	Abujabhah et al.^167^
Eucalyptus, papermill sludge, cow manure, poultry litter, and leaf waste	Pyrolysis at 400°C and 550°C			Lab scale experiment	High liming potential, nutrients, and carbon sequestration	Singh et al.^169^
Cornstover	Fast pyrolysis at 450°C and gasification at 700°C	Alfisol		Lab scale experiment	Increased CEC: biochar showed higher CEC (50.967 ± 1.890 cmol/kg) than the control soil (29.853 ± 3.098 cmol/kg), high O:C ratio	Lee et al.^170^
Eucalyptus	Pyrolysis at 350°C and 800°C	Sandy ultisol and clayey oxisol	20, 40, 80 g in 2 kg soil	Pot study	Lower temperature BCs were effective in increasing soil properties and plant growth.	Butnan et al.^171^
Papermill waste	Pyrolysis at 550°C	Ferrosol and loamy calcarosol	10 t/ha	Pot study	Reduced soil acidity and increased soil fertility and crop production (250% times higher than the control)	van Zwieten et al.^172^
Rice husk and shell of cotton seed	Pyrolysis at 400°C	Sandy loam soil	1kg in 20 kg soil	Pot study	enhanced soil moisture content, increased yield, (20% than the control), quality, and physiology of tomato in sandy loam soil under reduced irrigation	Akhtar et al.^173^
Hardwood and softwood	Pyrolysis at 500°C	Sandy loam soil	1kg in 20 kg soil	Pot study	Mitigation of salt stress in potato	Akhtar et al.^174^
Eucalyptus		Nitisol	4,8,12 t/ha	Lab scale experiment	Increased CEC (29.68% greater than the control), soil pH, yield (85.66% higher than the control), SOC, and available phosphorous, and enhanced fertilizer use efficiency	Abewa et al.^175^
Grass clippings, cotton trash, and plant prunings	Pyrolysis at 450°C	Alfisol	10, 50, 100 t/ha	Pot study	Increased the efficiency of nitrogen fertilizer (266%), and soil quality: soil pH, exchangeable cations, organic carbon	Chan et al.^176^
Corn stalk	Pyrolysis at 500°C for 6 h		2600, 5200, and 13000 kg/ha	Field experiment	Increased the pH, EC, and SOC. Enhanced the soil enzyme activities, and soil stability.	Tang et al.^177^
Sugarcane bagasse	Pyrolysis at 400°C–450°C	Clay laom	10, 15, 20 and 30 t/ha	Field experiment	Reduced the Cd toxicity (2.77–1.75 mg/kg), increased the soil fertility by increasing soil organic matter (30.01 g/kg from 20.46 g/kg) and available phosphorus (12.98 from 6.01 mg/kg)	Mohamed et al.^178^

The table discusses the various affecting parameters (feedstock material, production technique, pyrolysis temperature, and existing experimental conditions) prevailing during the soil amendment studies using BC. The table also highlights the different outcomes observed at the end of experiments.

As aforementioned, the properties of BC depend on the feedstock type and pyrolysis conditions. Singh et al.^169^ prepared BC from five different feedstock namely, eucalyptus, papermill sludge, cow manure, poultry litter, and leaf waste. The BC hence produced had high liming potential with minimal toxic elements and has high potential to sequester carbon and provide nutrients. Thus, it can be used to amend the degraded soil. In another study by Lee et al.*,*^170^ BC was produced under two different conditions, fast pyrolysis at 450°C and gasification at 700°C for soil amendment. Results showed that fast pyrolytic char had twice the cation exchange capacity (CEC) as that of gasification char indicating a higher O/C ratio as the major reason for such a mechanism. This was due to more hydroxyl, carboxylate, and carbonyl groups present in the fast pyrolysis char. The study indicated that controlling biomass pyrolysis conditions is crucial for producing desirable BC properties for soil amendment and carbon sequestration. Similarly, Butnan et al.^171^ explored the impact of pyrolysis temperature on soil fertility and plant growth (corn). Eucalyptus-based BC was produced at two different temperatures: 350°C and 800°C. Lower-temperature BCs were effective in increasing soil properties and plant growth. BC produced at higher temperatures demonstrated less efficacy due to high ash content than that produced at lower temperatures. High ash content in BC inhibited the plant growth. Meanwhile, Van et al.^172^ reported that papermill waste-derived BC produced through slow pyrolysis helped in reducing soil acidity due to its liming potential, thus increasing soil fertility and crop production.

BC also has possessed greater potential to ameliorate the negative effect of drought and salt stress in plants. For example, Saleem et al.^173^ examined the potential of BC on tomatoes under reduced irrigation. Results showed that the incorporation of BC enhanced the soil moisture content resulting in increased yield, quality, and physiology of tomato in sandy loam soil. The study suggested using BC as an effective measure for areas with limited water resources. Likewise, Akhtar et al.^174^ reported that BC helped in mitigating salt stress in potatoes. BC reduced the Na^+^ ions and soil bulk density leading to increased root growth, availability of K^+^ ions, and yield of potato suggesting BC could be a viable approach for soil amendment in saline soils. A study conducted by Wu et al.^179^ evaluated the effect of BC on saline soil. The results demonstrated that BC successfully lowered the soil pH, and exchangeable sodium percentage, and raised the soil organic carbon, available phosphorus, and cation exchange capacity.

Additionally, earlier studies have reported that BC has the ability to boost fertilizer use efficiency. In the study reported by Abewa et al.^175^ which evaluated the impact of BC on crop yield and soil properties, five different combinations of amendments were experimented; lime, BC, and N&P fertilizer. According to the study, BC improved the soil pH, CEC, soil organic carbon, and available phosphorus. BC combined with N&P fertilizer increased the crop yield suggesting that BC can significantly enhance the fertilizer use efficiency. Similarly, Chan et al.^177^ reported that BC can improve the efficiency of nitrogen fertilizer. They applied BC at three different rates, with and without nitrogen fertilizer to examine its potential on radish yield and soil quality in an Alfisol. It was found that BC enhanced the radish yield when applied at higher rates in the presence of nitrogen fertilizer. It also improved the soil quality by increasing the soil pH, exchangeable cations, and organic carbon, and decreasing tensile strength.

Conclusively, researchers have illustrated the capacity of BC to function as an eco-friendly sorbent in environmental applications, such as ameliorating soil health and mitigating soil contamination. BC demonstrated the potential to adsorb various organic and inorganic pollutants, increase soil fertility, improve soil properties such as pH, CEC, and microbial diversity, and retain nutrients, making it a viable tool for achieving sustainable development goals, particularly, SDG 15, which is focused on life on land. However, it is important to note that the BC properties vary depending on feedstock type and pyrolysis conditions. There is need for large scale application methods to understand the long term effects on soil health and crop productivity. Therefore, it is crucial to undertake extended field trials to offer more comprehensive insights into the evolution of biochar properties during aging, its enduring impact on soil properties, and the optimal timing for the re-application of various types of biochar. Further research is required to optimize BC production, integrate it with existing agricultural practices, and ensure its economic viability and environmental sustainability. Overall, BC holds significant potential to enhance soil health, increase crop yield, and provide a sustainable solution for soil and water ecosystem remediation.

### Mitigation of greenhouse gas emissions (SDG 13 - Organize climate action)

**Figure 5:**
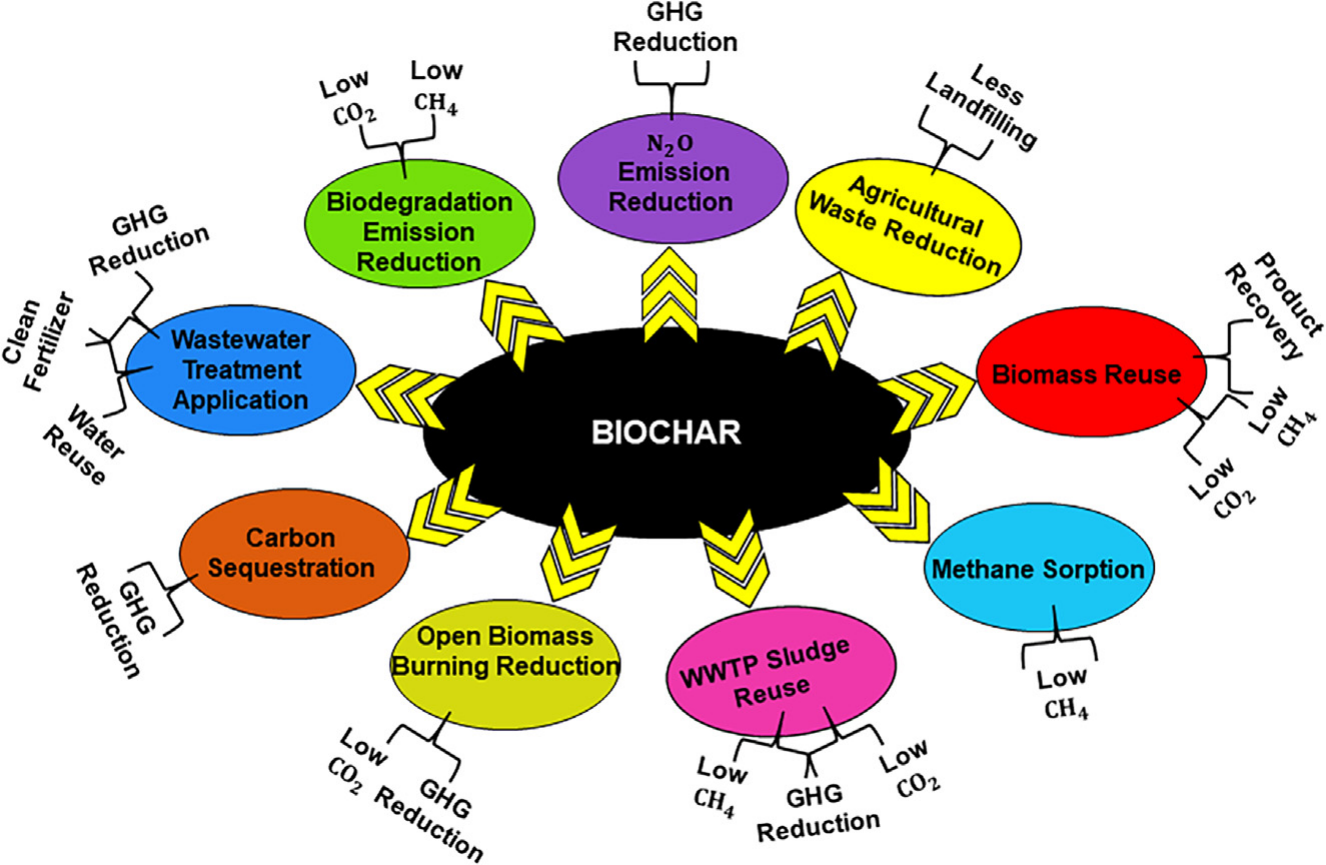
Application of BC for mitigating GHGs during soil amendment and wastewater treatment processes Utilization of BC in soil amendment supports the reduction of N_2_O, CO_2,_ and CH_4_ emissions, declines open biomass burning, decreases agricultural waste biomass, and simultaneously helps reutilize the biomass. In addition, using BC in the treatment of water treats the various pollutants, and reduces the emission of GHGs while it supplements water for reuse and clean fertilizer for plant growth as a result. So, the application of BC to soil and an aqueous environment apprehends positive impacts on the surroundings.

Global emission of greenhouse gases (GHGs) from developmental activities has become very alarming in a few decades. The current scenario of global warming will add a 1.5°C rise in global temperature by 2035, and 2.5°C by 2100. Further, envisioning the worldwide scenario, various reports demonstrate that seven countries (i.e., India, China, USA, Australia, Brazil, Canada, and Chile) emit more than 50% of global soil GHG emissions.^180^ Fossil fuel burning, farming/agricultural practices, deforestation, and urbanization are some of the major sources contributing to global GHG emissions. The GHGs released from different sources form a gaseous blanket in the troposphere which allows all the incoming solar radiation but captures all outgoing thermal/Infrared radiation producing a warming effect, hence, the term greenhouse effect has been coined. Consequently, the greenhouse effect then augments certain climatic variations in the atmosphere which simultaneously modulates the precipitation patterns, sea level rise, melting of glaciers, frequent floods, droughts, habitat fragmentation, biodiversity loss, and microbial activity (that causes several diseases) that affect the food security and human health in long run.^181^ As aforesaid, there exist different sources releasing GHGs which include agricultural practices that alone contributed to about 13.5% of GHG emissions and enhanced the anthropogenic GHG emissions by 81% globally between the period of 1961 and 2016.^182^ Arid and semi-arid parts of the world follow irrigated agricultural practices, which emit substantial amounts of GHG to the atmosphere. Rice cultivation, organic manure production/application, manure storage, agricultural residue fermentation/burning, and use of machinery are the practices of GHG emissions in farming. However, some practices of agriculture help in GHG reductions such as; carbon sequestration in the form of biomass, enhancing carbon sink into the soil, reduced microbial biomass decomposition, and reuse of agricultural residue instead of burning.^14^ Moreover, when these developmental activities limit the appropriate atmospheric pattern and climatic behavior, various government organizations have come into action to effectively combat such situations through different productive measures and approaches. Among these government organizations, as aforementioned, the UN general assembly formulated the 17 SDGs to resolve various issues existing in nature to uplift the life residing in the environment. Interestingly, these SDGs include one of the targets that dealt with climate actions (SDG 13) and further discussed 43% GHG emission reduction by 2030 and the commitment of net zero up to 2050. As a result, various materials, technologies, and processes are being developed to achieve SDG 13. To develop such materials, various research and experimental studies have been undertaken and thus, the viability of BC for such climate actions was identified (provided in Figure 5). Earlier studies have reported the applicability of BC for the reduction of different GHG emissions formed during the contaminant removal process from wastewater treatment and soil amendment purposes. So, further sections will provide insight into the efficiency of BC application in GHG mitigation during various treatment processes and associated mechanisms.

#### GHG mitigation during soil amendment

**Figure 6:**
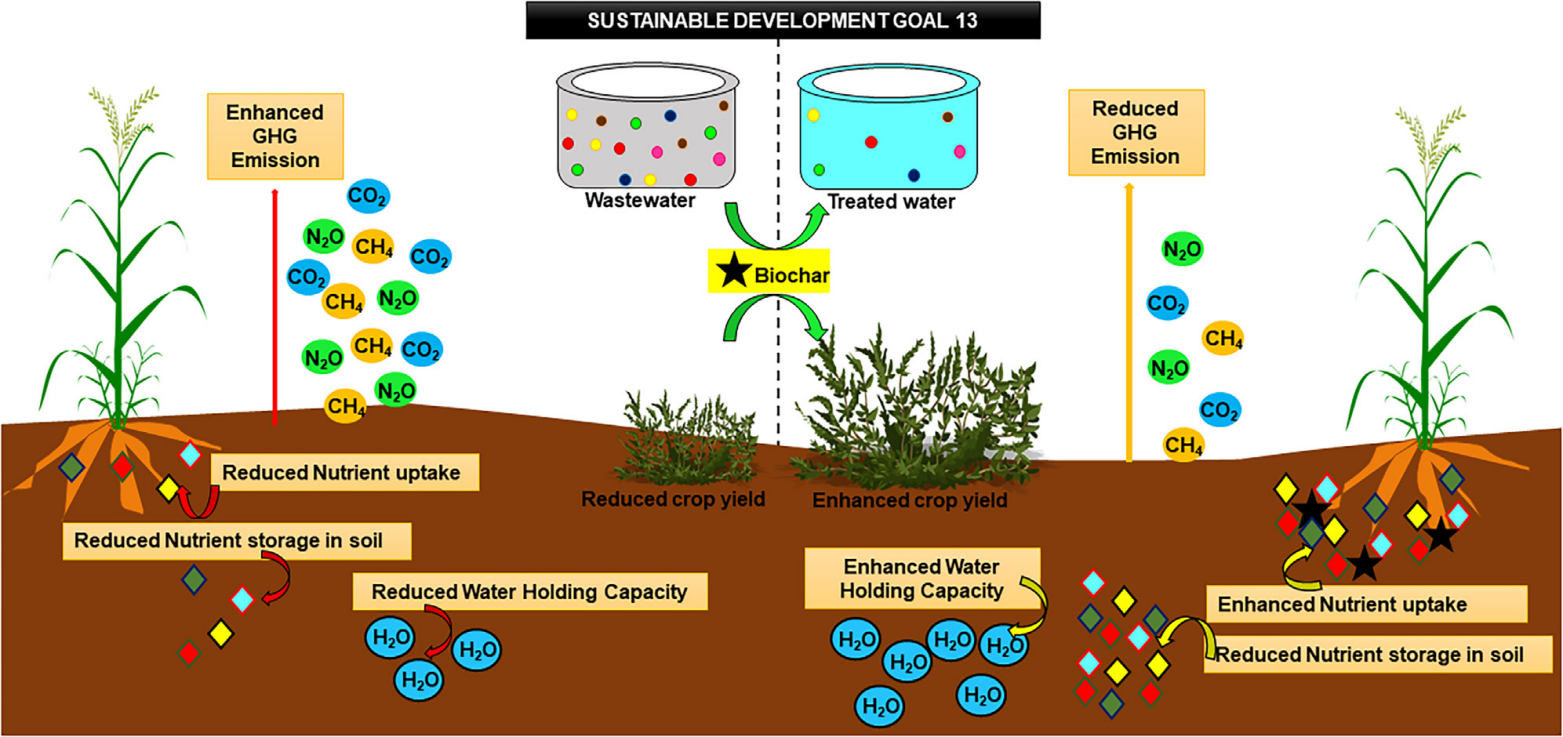
Application of biochar for soil amendment and simultaneous GHG mitigation Application of BC in soil enhances soil properties through improved nutrient uptake, storage capacity, and water holding capacity. Meanwhile, BC also reduces the emission of GHGs from soil and treats the pollutants from water, thus elevating the quality of air while enhancing the characteristics of soil and water. So, the utilization of BC in soil amendment and water treatment could subsequently amend in organizing climate action (SGD 13).

BC production from agricultural and other biomass has gained much attention due to its renewable properties, recalcitrant nature, strong carbon chain, high functionality, surface properties, and non-sophisticated processing.^14^^,^^183^ Such characteristics of BC make it a sustainable potential material to deal with climatic variations in different ways. Noteworthy, as some of the climatic variations are a part of agricultural activities, hence, the primary application of BC to agricultural fields would help mitigate part of the emitted GHGs (depicted in Figure 6). So, several research studies have experimented with the utilization of BC in agricultural fields which then participated in carbon sequestration, methanogen activity, and biological nitrogen cycling and accomplished successful GHG mitigation. Additionally, BC possesses significant water-holding capacity, nutrient capture, carbon storage, nutrient release, wastewater treatment, and pathogen reduction ability.^184^ Its application in soil has helped in enhancing carbon sequestration in a stable form after organic matter degradation. In a study, Windeatt et al.,^185^ estimated that 300 ton annual BC production could help in 0.5 billion ton carbon sequestration from CO_2_ emission per annum. This is approximately 1.5% of annual CO_2_ emissions. Similarly, Liu et al.,^186^ reported that carbon storage in the form of BC could help to reduce the annual CO_2_ emission by 0.3 billion tons. As per an estimate, 78% carbon containing BC, could store about 7–110 giga-ton carbon through a maximum 10,0000 kg per hectare application rate, on a 1411 million hectare stretch of the cropland.^16^^,^^187^ So, BC application in the soil makes a steady carbon availability due to its longer stability. Some studies have validated that the application of BC will capture more CO_2_ than its release from the soil environment.^188^^,^^189^ Yang et al.,^189^ elucidated that 41.02% of BC application to the soil will reduce the GHG (CO_2_) emission up to zero. Furthermore, BC addition with a 2% (w/w) application in the soil will avenge methane emissions close to zero.^190^ However, very limited information is available which details the efficiency of BC in controlling GHG emissions (such as methane and nitrous oxide). In a study by Liu et al.*,*^191^ the combination of slag + BC (8 + 8 ton ha ^−1^) application in a paddy field reduced the CH_4_ emission by 45%. The study was extended up to a duration of four years, where BC application had reduced up to 1/5^th^ of the total methane emissions from paddy fields with the simultaneous upliftment of rice yield to about 5%. The successive reduction of methane emission occurred due to BC application as it limited the growth of methanogenic archaea microbes and subsequently favored the growth of methanogenic bacteria.^192^ The aromatic structure of BC also acts as an electron acceptor and adsorbs dissolved organic carbon (methanogen substrate), hampering methanogenic activity and CH_4_ emission reduction. Additionally, soil amendment in paddy fields modulates the GHG emission with seasonal variation. For instance, Liu et al.^191^ observed fluctuations in CH_4_ emission with BC application in different seasons for soil amendment during flooding periods in paddy fields. Early rice season produced higher CH_4_ emissions than late rice season, possibly due to high methanogen activities developed through anaerobic conditions created by flooding.^193^ Continuous BC application for more than four years in paddy fields have reduced half of the annual methane emission and enhanced the crop yield by 13% annually. In contrast, it increased more than 1.5-folds of N_2_O emissions simultaneously.^191^ Furthermore, Jeffrey et al.,^194^ elucidated that BC application in flooded soil promoted the strength of the methane sink resulting in CH_4_ emission reduction as it promoted the activity of methanotrophs for methane reduction.^16^^,^^195^ In addition, BC enhances carbon retention for a longer period of time, along with the development of healthy soil (such as soil aerobic conditions, soil moisture, porosity, and vital microbes).^195^ This property helped in mitigating methane emissions and thus, the reduction of one among the GHG emissions.

Although BC reduces the CO_2_ and CH_4_ emissions with high crop yield and quality, it increases the N_2_O emissions by 150–190% annually.^196^ Previous research studies have reported that soil amendment with straw BC alone has enhanced the N_2_O emissions up to 43–78%.^197^ In contrast, many studies have also reported a reduction in N_2_O emission while using BC for soil amendment. For example, Wang et al.,^196^^,^^198^ reported a maximum of 34.1% and 20.5% of CH_4_ and N_2_O emission reduction respectively using combined slag and BC (8t Ha^−1^ each). Similarly, Lee et al.,^199^ mentioned that 20-ton ha^−1^ BC application in soil has reduced 33.2% higher N_2_O emission as compared to control setups. The improvement in N_2_O mitigation was apprehended to BC application that enhanced the soil organic carbon pool along with N_2_O emission reduction in different soil.^199^ Such phenomenon occurs as BC interacts with the nitrifying and denitrifying bacteria and inhibits complete nitrogen transformation affecting the reduction in N_2_O mitigation, released either from soil or water systems. Also, fertilization or composting promotes these metabolic reactions catalyzed in soil and contributes to higher N_2_O emission. So, BC addition enhanced the soil pH and promoted higher N_2_O emission ^191^^,^^200^ Additionally, the moisture content of the soil also affects the rate of N_2_O emission. High soil moisture is associated with anaerobic conditions, favoring denitrification, whereas low moisture promotes nitrification reactions. Brunn et al.^201^ estimated that about 8–23 times more N_2_O is generated under 80% moisture content than 40% moisture content in the soil. Yanai et al.^202^ conducted a laboratory experiment and found that 10% BC (charcoal) addition to soil has reduced 89% of N_2_O emission. Jia et al.^203^ found that BC application reduced the N_2_O emission up to a maximum of 82% in high moisture and high nitrogen-containing soil for vegetable production. Various studies have also reported that a higher amount of BC addition enhanced the mitigation of N_2_O emission up to a certain level depending upon soil types, while no change in N_2_O emission was observed at low BC addition (2–10%).^199^^,^^204^ Also, nitrate adsorption on the BC surface is also responsible for N_2_O emission reduction in soil.^205^ Biochar having a high C/N ratio could effectively adsorb more NH_3_ from soil, thus successively reducing the ammonia volatilization.^206^ The growing season of the crop also contributed to nitrous oxide emissions, as it required fertilizer (nitrite/nitrate) at different intervals of time. In the early crop growing period mixing of nitrogen with BC has enhanced the nitrification rates resulting in higher nitrous oxide emission. This emission also reduced with the advancement of the growing season of the crops, due to reduced denitrification.^207^ Edwards et al.,^207^ estimated that BC addition with nitrogen could reduce 37% of nitrous oxide emission, while the contribution of N_2_O emission increased to 41% without any nitrogen addition to soil.

Moreover, the surface properties of BC play a crucial role in driving these microbial reactions in nature. Thus, the raw material of BC, pyrolysis condition, pyrolysis temperature, modification in BC, and combined application of BC with other types of amendments are responsible for differential mitigation in GHG emission. In a study Jeffery et al.^194^ reported that BC prepared at 450°C–600°C promoted the methane source potential and reduced the methane sink potential on the soil application. However, the methane source strength has decreased and methane sink strength has increased after the BC application pyrolyzed at 600°C. Thus, the pyrolysis conditions of BC also affect the methane emission in soil. Furthermore, different biomass has different constituents in its composition. Pyrolysis produces specific types of surfaces and pores depending on raw biomass properties. More specifically, it brought changes in carbon matrix and particle size. In terms of methane sink and source strengthening, lignocellulosic biomass produced by BC enhanced the source and hampers the sink, especially for methane emission.^16^

Earlier experimental studies have mentioned that BC helped in enhancing crop yield, through improvement in plant and soil health. This was visualized in the form of healthy plant growth, root development, enhanced gaseous exchange through the plant, soil moisture, soil air, bulk density, and reduction in methanogen development. As fossil fuel and biomass burning release huge amounts of CO_2_ into the atmosphere, this part of carbon could be stored in the form of plant biomass. On the opposite side, traditional biomass burning will relocate the CO_2_ back to the atmosphere. So, conversion of these plant biomass in the form of BC could fix the carbon for a longer period. Structurally, BC is resistant to many biological, chemical, and physical degradation, which generates a fixed carbon pool available in the soil for a longer period of time.^25^ However, in terms of GHG mitigation, BC has shown significant emission reduction of CO_2_ and CH_4_, still it limited in terms of N_2_O mitigation. As BC inhibits the growth of methanogenic bacteria, the release of CH_4_ gets suppressed, while, in terms of N_2_O, BC interacts with the nitrogen-transforming bacteria and thus disrupts the nitrogen cycle thereby releasing N_2_O as a by-product. Also, the characteristics of BC absorb moisture leading to the existence of anaerobic conditions in the soil microclimate. Such nature of BC facilitates denitrification and simultaneous release of N_2_O. The reduction of these GHGs has been an essential component of SDGs and will surely play an important role in global warming reduction. So, the application of BC in the soil will efficiently help in GHG mitigation with various approaches. Likewise, when such BC material was applied for the treatment of various wastewater, it also favored the reduction of the emitted GHGs during the process. So, the further section encompasses the GHG mitigation strategy during wastewater treatment through BC application.

#### GHG mitigation during wastewater treatment

Fossil fuel combustion, deforestation, and agricultural emissions are major GHG emission sources. However, CO_2_, CH_4,_ and N_2_O emission also take place from swamps, drainage, septic tanks, wastewaters, and wastewater treatment plants (WWTP).^208^ Inadequate management of wastewater will lead to GHG emissions from many point and non-point sources. This makes the technique of wastewater treatment very important. WWTPs are the central facility for the treatment of wastewater, which also emits a substantial amount of GHG through its operational steps. WWTP operational units (such as biological and chemical), sludge dewatering, and sludge disposal are the main sources of GHG emissions. The emissions were in the form of CO_2_, CH_4_, and N_2_O as well. However, CO_2_ emissions from the WWTPs are not considered GHG by the Intergovernmental Panel on Climate Change (IPCC), claiming that the emissions are from fugacious biogenic sources. However, the contribution of anthropogenic CO_2_ in the GHG effect is higher than other minor GHGs due to high resultant atmospheric concentration.^209^ Many studies on WWTP emissions endorse the hypothesis that the emissions from WWTPs should not be ignored.^209^^,^^210^^,^^211^ Depending upon the influent wastewater property a municipal WWTP could emit about 5–1000 g of CO_2_ per cubic meter of wastewater.^212^ Activated sludge, trickling filter, rotating biological contractor, and up-flow sludge anaerobic blankets such as WWTP units were used for municipal wastewater treatments, which could produce CO_2_ and CH_4_. WWTPs produce huge amounts of sludge on a daily basis that contains greater concentration of organics and nitrogen in complex forms, which could be pyrolyzed in the form of BC. This carbonaceous BC will retain the carbon for a longer period, hence prohibiting its transfer to the atmosphere in the form of CO_2_ and CH_4_.^213^ BC production through sludge will suppress the N_2_O emission, which is considered a much better application of sludge instead of direct use in crop fields.^25^ Direct use of sludge as soil conditioner will trigger health and ecological toxicity. Although sludge-derived BC also represents some environmental hazards such as heavy metal leaching, some intensive research will promote its applications. For example, Fang et al.,^214^ produced the phosphorus-enriched BC from municipal sludge by regulating the temperature between 500°C and 600°C through microwave-assisted pyrolysis. Such BC contains high surface area and total phosphorus content that binds the available phosphorus and releases it into the soil slowly according to the requirement. These modifications could help derive phosphorous-based fertilizers from municipal sludge for field-scale and long-term applications. Suitable temperature range and residence time optimization will reduce the energy input and required carbon footprint.^209^

Further, several studies have incorporated BC produced from biomass as an adsorbent for wastewater treatment. BC used as a substrate matrix in bio-filter shows better nutrient retention from wastewater.^183^ It helps in pollutant breakdown and biomass generation, reducing CO_2_, CH_4_, and N_2_O emissions.^183^ A similar strategy is incorporated in constructed wetlands (CW) for the mitigation of GHG emissions using BC as substrate material during wastewater treatment. N_2_O is produced in limited nitrogen removal conditions of CWs.^215^ BC helps in the regulation of microbial composition in CW systems, controlling GHG emissions. BC addition in the CW system as a substrate has reduced GHG fluxes, which are produced during the biological decomposition of wastewater. In order to do this, Guo et al.,^216^ have studied a CW system with different chemical oxygen demand/nitrogen ratios and found that BC containing CW has reduced the global warming potential from 18.5% to 24% of N_2_O and CH_4_ respectively. About 21 Ktaeq of CO_2_ could be captured through the BC production from 100 tons/day sludge generating WWTP with 65% BC yield,^209^ as 0.9 kg of GHG emission reduction takes place from per kg of BC production. Meanwhile, BC surface property could entrap the nitrogenous compounds of wastewater in CW systems as well. Recent studies highlighted that BC addition to gravel or ceramsite-based CW shown enhanced N_2_O reduction.^217^ Also, increasing the BC proportion in CWs will reduce the N_2_O emission from CWs,^216^ as BC supports more *nos*-Z promoting effective nitrification-denitrification and reducing the N_2_O emission.^218^ CWs itself produced biomass in the form of plant (aquatic macrophytes such as *Canna indica* and *Glyceria maxima*) and microbial biofilm apprehending better CO_2_, CH_4_, and N_2_O emission reduction potential.^3^^,^^12^^,^^219^ Microorganisms and plants in CW work in collaboration, where the organic matter of wastewater is being converted into carbon-containing substances and inorganic nutrients (such as nitrate and phosphate).^220^ This carbon-based compound is utilized by microbes for biomass generation and inorganic nutrients will be used by plants. Thus, CO_2_ and CH_4_ convert into biomass instead of getting released as GHGs. The macrophytes will capture atmospheric CO_2_ for photosynthesis which was released from wastewater. Moreover, protein, ammonia, nitrite, and other organic nitrogen compounds will convert in the form of nitrate. This nitrate will be used by the macrophytes of CW, limiting the emission of N_2_O.^221^ On the other hand, BC derived from sludge, biomass, and algae could also be used as a substrate matrix in CW systems. BC helps to promote biological metabolism which acts on CH_4_ reduction, carbon sequestration, and reduction in N_2_O flux through substrate matrix, flora, and fauna in CWs. Additionally, BC could adsorb the N_2_O generated in CWs for biotic and abiotic consumption. Ji et al.,^222^ reported lower methane emission from BC containing CW than ceramsite material, attributing high methanogens/methanotrophs in CW bed. High methane consumption will be observed due to good oxygen conditions produced through the porous BC structure.^222^ Such performance of BC is associated with its various characteristics. For example, BC surface properties especially free radicals, surface charge, phenolic and quinone contents interplay in CWs will lead to varied emission reduction.^183^ BC with some alkaline properties will promote CO_2_ dissolution and precipitation. But macrophytes require CO_2_ for their growth and subsequently utilize the released CO_2_ during photosynthesis.^216^

Overall, wastewater and simultaneously WWTPs could be a source of GHG emissions as it could emit CO_2_, CH_4_, and N_2_O-like gases. Sludge generation and other biological units of WWTP such as the activated sludge process, trickling filter, and rotating biological contractor contribute in GHG emissions through biological metabolic reactions. Similarly, CWs also contribute to GHG emissions from biological reactions. However, BC production and application in soil amendment and wastewater treatment have mitigated CO_2_, CH_4_, and N_2_O emissions. However, BC production from sludge and its applications such as soil amendment and wetland substrate matrix needs more scientific intervention and validation. Although it facilitated the reduction of GHG emissions, the adverse ecological effects are still vaguely identified. All types of natural and engineered wetlands play the role of carbon sinks if operated and adequately managed with BC. As proposed by Mitsch et al.,^223^ about 118 g-C m^−2^y^−1^ average carbon is being sequestered by wetlands which constitutes a maximum 8% of the whole terrestrial portion of the earth. Also, wetlands help in maintaining biodiversity, recreation, wastewater treatment, flood control, and GHG emission reduction. These properties of wetlands will help partially in many goals and targets associated with SDGs. So, the use of BC containing CW will reduce GHG emissions fulfilling SDG goal 13 of climate action. Still, more intensive research is highly essential to vitally inculcate BC as a suitable material for upscaling the natural environment and fulfilling the SDGs.

### Bioenergy generation (SDG 7- affordable and clean energy)

The application of BC for energy generation using biomass has been extensively used for various approaches. The thermochemical and biological conversion of BC helped in producing bioenergy through several pathways. In the thermochemical pathway, heat and temperature are provided to biomass and the resultant product helps for building energy storage devices such as supercapacitors, electrodes for bioelectrochemical systems, and many more.^224^ While, in the biological pathway, microbes decompose (through anaerobic reactions and fermentation) the biomass for the production of biofuels such as bio-oil, biodiesel, bio-hydrogen, ethanol, and so forth.^225^ The BC, thus formed from these technologies is cost-effective and consists of several viable characteristics such as porosity, SSA, and so forth*.* that formulated a pathway for its further experimental research and application in industries for bioenergy production.^226^ Currently, the quality of BC has been of immense importance for which various studies have modulated BC properties through activation technologies (physical and chemical activation) and enhanced the quality of BC according to the desired application.^227^ As a result, nano-composites of BC, such as functional, magnetic, and nano-metal hydroxide/oxide are essential for future research.^224^ This application of BC coincides with SDG 7 listing affordable and clean energy for environmental applications. BC offers a sustainable option for producing bioenergy for various environmental applications. So, with respect to such applications, BC and its modifications for sustainable energy generation have been discussed in further subsections.

#### Supercapacitors

Supercapacitors (electrochemical capacitors and ultracapacitors) are devices required for storing energy. The wide application of supercapacitors has been extended because of their effective dynamics for charge transfer, pulse power supply, expanded life cycle, simple working principle, and high power density.^228^ As supercapacitors serve as uninterruptible sources of power, they have been extensively explored in various power grid stations, electrical devices, and electrical vehicles. Earlier studies have reported the fabrication of these supercapacitors using various carbon materials, metal oxides, metals, chalcogenides, and their integrated materials.^229^ Also, studies have investigated activated carbon, graphene, and carbon nanotubes as efficient supercapacitor materials due to their high SSA, porosity, and many other factors. Additionally, such materials have a reduced impact on the environment and are economically feasible.^229^ Previously, several experimental studies have reported similar properties in various BC materials, based on which the design and fabrication of different supercapacitors have been explored. For such purpose, different materials derived from various sources such as agricultural residues, industrial by-products, woody biomass, and so forth*.* have been used for producing BC.^229^ For example, Mu et al.^230^ incorporated bean curd which was activated using K_2_CO_3_ and utilized as a supercapacitor electrode. The obtained results exhibited an energy density as high as 11.9 W h/kg at a current density of 0.1 A/g in the presence of 1 M H_2_SO_4_ solution. A highly stable capacitance performance for 10,000 cycles was achieved using two symmetric capacitors. Additionally, it was also observed that a 10 wt. % K_2_CO_3_ concentration among the selected range of 0–15 wt. % was highly suitable for the enhanced rate of performance. Similarly, Zhang et al.^231^ used wheat bran activated with NaOH which resulted in a gravimetric capacitance of 294.3 F/g at a current density of 0.5 A/g. Simultaneously, the material performed a stable capacitance performance for 50,000 cycles with a cyclic life of 94%. Likewise, Wu et al.^232^ developed microrods from *Albizia* flowers and activated them with KOH. The microrods depicted a higher specific capacitance of 406 F/g at a current density of 0.5 A/g in KOH (6M) electrolyte. Further, the same material provided a power density of 429 W/kg and an energy density as high as 26.3 W h/Kg. So, it could be predicted that the surface properties of BC, i.e., structural, functional, and morphological characteristics are the primary factors responsible for the capacitive performance taking place. Moreover, the nature of the biomass material used, thermal conditions, and the fabrication method also play vital roles. Studies have also incorporated various modification processes such as activation, composite film fabrication, metal/metal hydroxide/metal oxide loading, and nitrogen-sulphur doping that favored enhancement in the electrical performance of the material. Still, an in-depth investigation for obtaining such sustainable and cost-effective BC material is highly essential. Also, future interventions are yet to be discovered for enhancing the efficiency of the developed material incurring less energy input and operation facility.

#### Bioelectricity generation in electrochemical systems

A typical electrochemical system consists of a fuel cell and an electrolysis cell. Between these two systems, the use of fuel cells has widely been studied for bioelectricity generation and treatment of wastewater. A typical microbial fuel cell (MFC) consists of two chambers, anode and cathode, having anaerobic and aerobic conditions in nature.^233^ Both chambers were separated by a proton exchange membrane and consisted of electrodes connected externally by a resistance. The flow of electrons occurs from the anode to the cathode to complete the redox process and simultaneously generate electrical energy.^234^ So, for this purpose, various studies have reported the use of different electrode materials, such as graphite, platinum, carbon cloth, carbon brush, and so forth*.* to enhance the power output and the pollutant treatment efficiency of the system.^8^ However, these electrodes prevailed with various drawbacks such as high cost, scarce availability, and so forth*.* So, when studies have experimented with BC for such purposes, it was observed that BC is cost-effective in nature and easily available. Additionally, as aforementioned, BC are highly sustainable, and environment-friendly, possesses high porosity, SSA, and many more characteristics, these properties of BC make it a suitable candidate for such activities. As a result, various studies have incorporated BC as electrode material in MFC to enhance the performance of the system. For example, Hung et al.^235^ used electrode material derived from activated carbon of coffee waste that obtained a power density of 3927 mW/m^2^, while the commercially available activated carbon resulted in a power density of 975 mW/m^2^. Likewise, Yuan et al.^236^ reported use of sewage sludge and coconut shell BC produced a power density of 969 ± 28 mW/m^2^, which was 2.4 times greater than that of graphite and Pt electrode. Similarly, Wang et al.^237^ used corn-straw BC produced at 500°C (BC500) and 900°C (BC900) and BC modified with KOH (BAC). The results demonstrated that the power density of the BES system was enhanced by 92%, 56%, and 17% using BAC, BC900, and BC500 respectively as anode electrodes., the power density was enhanced by 4.8, 5.2, and 3.1 times using BAC, BC900, and BC500 respectively as cathode electrodes with respect to carbon felt. Overall, it could be withdrawn that using BC as electrode material could help enhance the energy generation efficiency sustainably. Still, more research is essential to study the use of BC and its composites that could be used as electrode material for energy generation and associated sectors responsibly.

#### Other applications

Considering the various energy generation sectors, earlier studies have reported the extensive application of BC for various other purposes such as the production of biofuel (bio-oil and biodiesel),^238^ carrying out water splitting mechanism,^239^ serving as a catalyst during biohydrogen production^240^ and supports conversion of methane to hydrogen^241^ as well. For example, Ozcimen et al.^242^ produced bio-oil using BC of different biomass materials (Apricot stone: AS, Hazelnut shell: HS, Grapeseed: GS, and Chestnut shell: CS). The result demonstrated that the BC (AS: 30.76, HS: 29.08, GS: 26.73, CS: 25.86) obtained from these materials had higher calorific value followed by the bio-oil (AS: 22.72, HS: 26.79, GS: 29.76, CS: 26.72) products and then by the raw materials (AS: 19.28, HS: 18.33, GS: 20.51, CS: 15.49). Likewise, Zhao et al.^243^ utilized Pomelo peel BC activated using K_2_CO_3_ for synthesizing biodiesel through a transesterification reaction. The study resulted in a biodiesel (fatty acids methyl ester, FAME) yield of 98% and worthwhile, the material expressed a better stability performance (>82%) though being used repeatedly for 8 times. Similarly, Bhatia et al.^244^ synthesized biodiesel from discarded cooking oil by incorporating waste cork (*Quercus suber*) BC as a catalyst. The results demonstrated that the BC material synthesized at 600°C depicted the highest FAME yield of 98% at an alcohol-to-ratio of 25:1. Further, Xia et al.^239^ used BC obtained from *Camellia japonica* flowers for electrode material in electrochemical reactions and doped with sulfur for effective water splitting reactions. The study suggested that during the water-splitting reaction, the electrode had a highly stable performance for both oxygen and hydrogen generation associated with the over-potential value of 362 and 154 mV respectively, at a current density of 10 mA/cm^2^. However, when the production of hydrogen is considered using BC, Yao et al.^245^ produced hydrogen from biomass gasification by applying BC of different materials (cotton and rice) as a suitable catalyst. It was observed that cotton-char (92.08 mg/g and 64.02%) outperformed rice-char in terms of H_2_ production. Moreover, Kundu et al.^246^ developed carbon-encapsulated iron nanoparticles for the decomposition of methane and the simultaneous formation of hydrogen. The study compared the efficiency between two cellulose BC prepared at 700°C and 800°C, where BC prepared at 800°C outperformed the other material with a higher conversion efficiency of 95.7% that reduced to 36.8% within a duration of 180min.

Conclusively, if used efficiently and sustainably, BC can be incorporated as an effective material having the potential for bioenergy generation. The surface properties of BC associated with further modification/activation amended better results during energy generation. Moreover, it is also observed that production temperature and feedstock type vitally drive the activity of BC. As a result, more focus should be enlightened on deriving effective BC material with reduced energy input and monetary investments. Also, the utilization of spent over BC (incorporated during wastewater treatment and soil amendment) could be analyzed for future studies. Additionally, the composites of BC should also be taken into consideration during energy generation applications, as it will reduce the cost incurred in using expensive materials. Still, more research study is yet to be focused in this field for extensively analyzing the applicability of BC for other bioenergy applications.

## Limitations and future prospects

BC has emerged as a potential material to fulfill sustainable developmental goals through its diverse properties.^247^ Partially, BC fulfills many SDG goals, but more specifically it contributes to SDG goal 6 (clean water and sanitation), 7 (Affordable and clean energy), 13 (Climate action), and 15 (life on land). BC applications for different purposes have addressed many social, environmental, and economic problems. Still, it faces various technical, financial, regulatory, environmental, and socio-economic barriers. BC production technologies should be well documented in terms of cost, surface properties, production emission, shelf life, and thermal condition.^247^ Much literature are available on these conditions, but well-documented studies/reports are still insufficient. For instance, different raw biomass for BC production required different thermal conditions for the production and incurred high costs based on availability. Such varied thermal conditions will produce BC with different ash contents, but their subsequent effects on soil, water, and other applications needs further research.^171^ Moreover, simple synthesis methods such as ball milling and co-pyrolysis methods should be studied and developed scientifically.^17^ Unplanned and poorly developed pyrolysis plants would release harmful air pollutants to the atmosphere leading to greenhouse gas emissions and climate change affecting SDG goal 13 of climate action. Meanwhile, the BC generated during this process could also cause various repercussions (human health hazards) to the associated workers/farmers which should be investigated and validated prior to its production.^247^ As a result, regulatory standards and specific application requirements mitigating those issues should be designed and developed efficiently but such processes might demand more cost and monetary inputs.^248^ Furthermore, BC application to soil would also enhance the growth of weeds and produce an inhibitory effect on soil aging.^248^^,^^249^ A high rate of BC application in the soil at the rate of 15 t/ha will increase the growth of weeds up to 200 times.^250^ Moreover, aged BC in soil will exert negative effects in soil by hampering the earthworm and fungus growth.^251^ Additionally, BC has produced distinct results (positive and negative) with different species and plant parts on soil application.^248^^,^^252^ Further, the pollutant adsorption capacity of BC in soil is limited to certain groups. For instance, BC application had not hampered dichlorodiphenyltrichloroethane pesticide uptake in the soil.^253^ Bio-based production of fertilizers, chemicals, and other by-products could be used for soil conditioning and smart farming to uplift crop productivity and maintain the soil resource meeting SDGs.^17^ So, BC application to the soil medium should be clearly monitored and also release of BC and associated contents into the water bodies (underground/surface water) through soil should be regularly assessed to prohibit any futuristic reluctant activity.^254^ Moreover, it was observed that BC application in wastewater treatment have reduced organics, metals, nitrogen, and sulfur with its surface properties and helped in macrophyte growth in constructed wetlands (CWs) and other wastewater treatment systems.^183^ This helped in wastewater treatment and reduced the land footprint contributing to SDG goal 15. In contrast, the adsorption property of BC could remove essential nutrients such as iron and nitrogen from the soil, affecting plant growth.^255^ This could develop a competitive scenario between BC and plants for nutrients.^248^^,^^256^ Likewise, when the utilization of BC for bioenergy generation is considered, careful investigation should be carried out as a lack of proper BC material would degrade the energy generation tendency of the system. Also, modification, doping, and activation of the BC material should be analyzed prior to assessment, as it would facilitate the effective production of clean fuel for various purposes. So, to overcome such limitations of BC various other modification strategies or developing composites with other materials should be taken into consideration. Such methods could help in preparing BC material that enhances the bioenergy generation tendency with reduced cost and enhanced sustainability. These modifications will also favor limiting the drawbacks of BC with regard to the release of N_2_O into the environment. Possibly, modified or composite BC would accomplish nitrogen transformation processes successfully leading to a decline in N_2_O release. Also, the desorption processes should also be carried out to understand the longevity of the BC material sustaining the pollutants within it. Further, Kinetics of the pollutant removal should be highlighted and explicitly studied to get a clear insight into the existing mechanisms and the prevailing reaction pathway. This will also guide in making required configurational changes for producing efficient BC material. However, during this process, the economy of BC should be deliberately examined as the developed material should be cost-effective in nature. Incurring higher costs for preparing the BC would reduce its large-scale potential applications. Meanwhile, comprehensive risk assessment, toxic effects, and safe application ratio/rates of BC are also highly important for long-term use. Thus, BC with greater surface properties has been proven as a carbon-neutral material for achieving different SDGs. So, materials such as BC will be the future of a sustainable society and self-reliant ecosystem, if incurred vigilantly. Still, there exists a vital need for profound and extensive studies relating to BC production and field-scale application.

## Conclusions

The sustainable characteristics of BC stand as a beneficial feature that strengthens and uplifts the different spheres of the environment, i.e., air, water, and soil. The attained feasibility of modified and unmodified BCs for treating different aqueous mediums, enhancing soil properties, remediating GHG release, and effective bioenergy generation acclaims the achievement of various SDGs. The efficiency of most of these developed BC materials was much higher (>80–90%) and their performance vitally depends on different parameters such as the initial concentration of pollutant, pH of solution, adsorbent dosage, and many more. For such phenomena to occur, hydrogen bonding, electrostatic attraction, and π-π bonding primarily come into action to enhance the remediation process. Although these BC materials supplement better performance for pollutant abatement, still they avail some limitations that drag attentive measures. During GHG mitigation, though BC reduces the release of CH_4_ and CO_2_, it compels the release of N_2_O. More focus needs to be driven for accounting the occurrence of such processes. Also, future use of spent BC is of prior concern, as the post-treatment BC material consists of different harmful pollutants which need further treatment. However, the nutrient-enriched and treated BC could be used for futuristic agricultural applications. Further, more findings highlighting the distinct composites of BC with other metals and minerals could demonstrate advanced energy applications and uplift remediation strategies. Meanwhile, the knowledge and compilation of underlying kinetics taking place during the pollutant removal mechanism also need an in-depth analysis, as these kinetic studies propose better reaction pathways and provide suitable practical implications. Overall, such sustainable applications of BC apprehended that these materials could effectively facilitate in achieving the primary goal of SDGs, i.e., to uplift man and the environment through ecological solutions. If such materials having high SSA, greater porosity, and abundance of functional groups could be modified and utilized properly, it would enhance the possibility of attaining other SDGs in the immediate future. Also, more research and development for identifying and designing similar materials with improvised characteristics should be carried out as sustainable solutions for uplifting the environment and society securely.

## Acknowledgments

Ashmita Patro would like to acknowledge the UGC-NET fellowship provided by 10.13039/501100001501University Grants Commission, India for assisting financial support. She is also thankful to AcSIR, New Delhi, and CSIR-Institute of Minerals and Materials Technology (CSIR-IMMT), Bhubaneswar, India for helping her to carry out Ph.D. work. PKS would like to thank the Department of Science and Technology (Government of India) for providing support to this work through DST-SERB Core Research Grant (CRG/2021/002567).

## Author contributions

Ashmita Patro: Conceptualization, writing, design of the work, data collection and interpretation, drafting the article. Saurabh Dwivedi: writing, drafting the work, data collection, and editing of the article. Anjali Thakur: writing the work, data collection, and compilation. Dr. Prafulla Kumar Sahoo: Conceptualization and design of the work, supervision, review and critical revision of the article, and final approval of the version to be published.

## Declaration of interests

The authors have no relevant financial or non-financial interests to disclose.
